# Selection Gain of Maize Haploid Inducers for the Tropical Savanna Environments

**DOI:** 10.3390/plants10122812

**Published:** 2021-12-19

**Authors:** Abil Dermail, Sompong Chankaew, Kamol Lertrat, Thomas Lübberstedt, Khundej Suriharn

**Affiliations:** 1 Department of Agronomy, Faculty of Agriculture, Khon Kaen University, Khon Kaen 40002, Thailand; abildermail@gmail.com (A.D.); somchan@kku.ac.th (S.C.); 2 Plant Breeding Research Center for Sustainable Agriculture, Khon Kaen University, Khon Kaen 40002, Thailand; kamol9@gmail.com; 3 Department of Agronomy, Iowa State University, Ames, IA 50011, USA; thomasl@iastate.edu

**Keywords:** *Zea mays*, doubled haploid, haploid induction rate, *R1-nj* marker system, modified ear-to-row selection

## Abstract

Lacking elite haploid inducers performing high haploid induction rate (HIR) and agronomic performance is one of fundamental factors hindering the rapid adoption of doubled haploid technology in maize hybrid breeding, especially under tropical savanna climate. Breeding haploid inducers for specific agro-ecology, thus, is indispensable yet challenging. We used temperate inducer Stock6 as genetic source for haploid induction ability and eight tropical maize genotypes as principal donors for agronomic adaptation. Three cycles of modified ear-to-row with 5% intra-family selection were applied in a population set of 78 putative haploid inducer families emphasized on agronomic performance, *R1-nj* anthocyanin intensity, and inducer seed set. Genetic gains, variance components, and heritability on given traits were estimated. Hierarchical clustering based on five selection criteria was performed to investigate the phenotypic diversity of putative families. Cycle effect was predominant for all observed traits. Realized genetic gain was positive for HIR (0.40% per cycle) and inducer seed set (30.10% or 47.30 seeds per ear per cycle). In this study, we reported the first haploid inducers for regions under tropical savanna climate. Three inducer families, KHI-42, KHI-54, and KHI-64, were promising as they possessed HIR about 7.8% or 14 haploid seeds per tester ear and inducer seed rate about 95.0% or 208 inducer seeds per ear. The breeding method was effective for enhancing the seed set and the expression of *R1-nj* anthocyanin marker of inducers, yet it showed a low effectiveness to improve haploid induction rate. Introgression of temperate inducer Stock6 into tropical gene pool followed by phenotypic selections through modified ear-to-row selection on inducer seed set and *R1-nj* marker did not compromise the agronomic traits of tropical inducer families. Implications and further strategies for optimizing genetic gain on HIR are discussed.

## 1. Introduction

Hybrid cultivars account for major maize acreage due to their advantages including heterosis [1], high yield, and uniformity [2]. To ensure affordable hybrids for sale, elite inbred lines are the main prerequisites in routine maize hybrid breeding. Doubled haploid (DH) technology has significantly contributed to the improved production of maize inbred lines as it shortens the time required to achieve 100% homozygous lines from 6–8 selfings by conventional breeding to at least two generations [3]. The in vivo haploid induction system is currently preferable in maize since it takes much less time to produce lines with sufficient homozygous level [4] compared to the in vitro system that is high genotype dependence and costly [5].

As an integral part of in vivo DH technology, the maternal haploid induction system requires haploid inducers assigned as pollinator to generate haploidy [6]. The number of induced haploids per cross is considered as haploid induction rate (HIR). The first maize haploid inducer Stock6 had a 2.3% HIR [7], and subsequent improved inducer lines have been developed such as ZMS [8], KEMS, KMS [9], MHI [10], CAUHOI [11], RWS [12], PK6 [13], UH400 [14], PHI-3 [15], and BHI306 [16] with HIRs exceeding 10%. These Stock 6-driven haploid inducer lines belong to temperate inducers, likely exhibiting poor adaptation to the tropics [17]. Chaikam et al. [18] currently released 2GTAILs as the second-generation of tropically adapted inducer lines possessing 13.1% of HIR and good adaptation to both tropical and subtropical environments. Thailand has a typical tropical savanna climate with a more pronounced dry season and short but extremely rainy wet season [19]. To the best of our knowledge, no haploid inducer is available that is specifically adapted to this region.

Haploid induction rate of temperate backgrounds has been reported to be heritable, and additive effects predominant for HIR [20,21]. HIR is under polygenic control [21] and currently two cloned genes underlying HIR have been reported namely *mtl* [22] and *zmdmp* [23]. This trait is also sensitive to contamination with non-inducer pollen that has selection advantages over pollen of inducers [6]; thus, more attention should be paid to perform proper selection for HIR as one of major objectives in breeding haploid inducer lines. The reliability of selection method can be determined as genetic gain over consecutive breeding cycles [24]. Ear-to-row selection firstly introduced by Hopkins [25] allows breeders to do selection, seed harvest, and evaluation of progeny rows representing single plants. Modified ear-to-row selection resulted in positive selection gains for maize grain yield ranging from 5.3% to 13.6% [26,27,28]. However, no respective study has been reported for genetic gain of HIR and related traits by this method in tropical inducer development. This study aimed (i) to estimate the genetic gain of putative haploid inducer families across three cycles of modified ear-to-row selection on inducer seed set and kernel *R1-nj* marker intensity, (ii) to assess the correlated response of HIR and agronomic traits as an indirect response to selection on given selection criteria, and (iii) to investigate the phenotypic diversity of putative families on given selection criteria. Information obtained in this study will elucidate whether and to which extent phenotypic selection based on *R1-nj* enhances haploid induction ability and inducer seed set as major criteria for breeding haploid inducer lines.

## 2. Materials and Methods

### 2.1. Plant Materials and Establishment of Base Populations

A temperate haploid inducer Stock6 and eight tropical corn genotypes including three waxy corn open-pollinated varieties (KND, TL, and TB), two sweet corn breeding lines (101L and WST), and three commercial field corn hybrids (Pacific339, NS3, and NSX) were used to generate a base population. Stock6, a temperate maize genetic stock with 2.3% of HIR [7], was assigned as founder parent. KND, TL, TB, 101L, WST, Pacific339, NS3, and NSX assigned as principal donors are non-haploid inducer genotypes with good plant stand and tropical adaptation. KND, TL, TB, 101L, and WST are developed by the Plant Breeding Research Center for Sustainable Agriculture, Khon Kaen University, whereas NS3 and NSX are developed by the Nakhon Sawan Research Center, Thailand. Pacific339 is a commercial field corn hybrid from Pacific Seeds Thailand.

**Figure 1:**
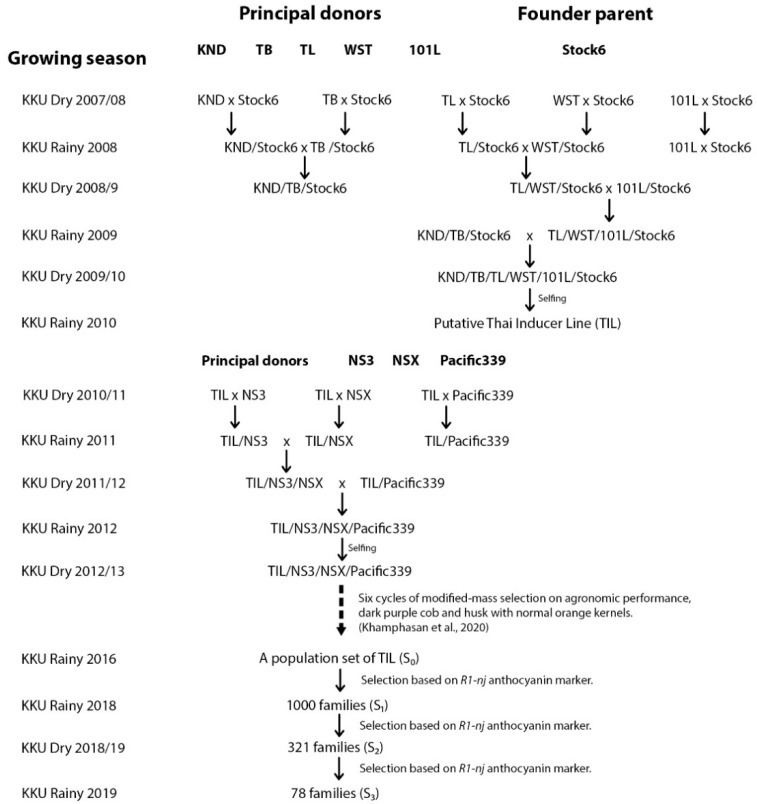
Breeding framework to establish a population set of 78 putative haploid inducer families (2007–2019).

Intercrosses were made between Stock6 and each of non-inducer genotypes (101L, WST, KND, TL, and TB) (Figure 1). Six cycles of modified mass selections were carried out regarding plant stand, flowering synchrony, dark purple stem, husk and cob, and orange kernels [29]. Two more cycles of selection (S_1_ and S_2_ families) were performed to identify seeds expressing *R1-nj* anthocyanin markers both in the endosperm and embryo. Finally, 78 S_3_ families performing good tropical adaptation were obtained in the rainy season of 2019.

### 2.2. Population Improvement and In Vivo Haploid Induction

**Figure 2:**
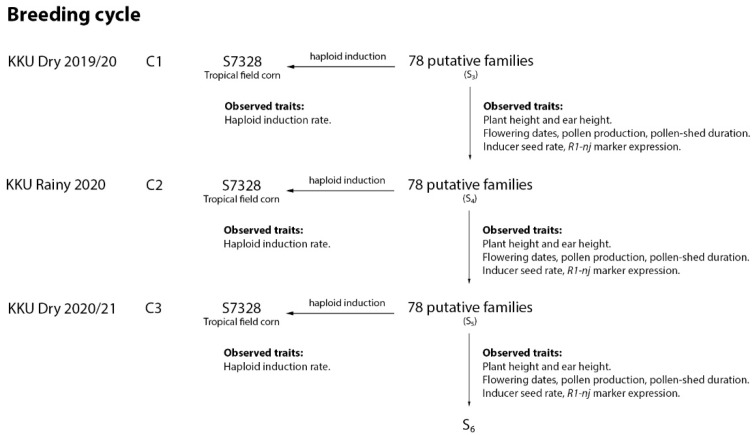
A two-way scheme of haploid induction (horizontal) and line purification (vertical). C1, C2, and C3 represent breeding cycle 1, cycle 2, and cycle 3, respectively.

A population set comprised of 78 S_3_ families of putative haploid inducers (Table S1) was subjected to a randomized complete block design (RCBD) with two replications at Agronomy Field Crop Station, Khon Kaen University, Thailand (16°28′27.7” N, 102°48′36.5” E; 190 m above sea level). Each family plot consisted of 2 rows of 5 m length, plant spacing was 75 cm between and 25 cm within rows, with 40 plants per plot. This design applied for all three consecutive breeding cycles in the dry season 2019/20 (C1), the rainy season 2020 (C2), and the dry season 2020/21 (C3). Improvements of all putative families were emphasized on agronomic performance, *R1-nj* anthocyanin intensity, inducer seed rate (ISR), and HIR (Figure 2).

Modified ear-to-row with intra-family selection was applied through three selection steps: (1) On-field selection using single plant basis regarding good plant stand and uniformity. About 15–20 plants per plot were selected, and the pollen was bulked for dual purposes, namely, haploid induction and line purification (Figure 2). (2) Inducer ear selection at harvest stage. About ten ears derived from self-pollinated plants (step 1) per family plot were selected regarding *R1-nj* marker expression on the crown endosperm and visual seed set. (3) Final single ear selection based on inducer seed rate and *R1-nj* marker expression. The top two ears per family plot were separately kept for further evaluation in the next breeding cycles. Therefore, selection intensity of each putative inducer family in each breeding cycle was 5%.

Haploid induction was performed to estimate HIR. A tropical semi-dent field corn hybrid cultivar S7328 was assigned as female tester. Released by Syngenta Co., Ltd., this tester is popularly grown by farmers in Thailand as it is drought-resistant and has large seeds with orange pericarp pigmentation. Bulk pollen of ten inducer plants per family plot was used to pollinate ten tester plants. To ensure flowering synchrony for haploid induction, three staggered planting dates of tester genotype with seven days interval were carried out, namely, 14 days before planting inducers, 7 days before planting inducers, and on the same day as planting inducers. The crop field management followed the Department of Agriculture, Thailand, recommendations [30] including fertilization, irrigation, and pest, disease, and weed control.

### 2.3. Field Data Collection

During the vegetative stage, all families were observed on plant stand by 5-scale rating on overall performance of all plants within a plot regarding plant vigor, leaf erectness, and stem size at V8 stage or around 1 month after sowing (MAS), ranging from score 1 (excellent plant stand with good vigor, erect leaves, thick stalk, and pests and diseases-free) to score 5 (poor plant stand with weak vigor, horizontal leaves, thin stalk, and susceptible to pests and diseases). During the reproductive stage, all families were evaluated on (i) anthesis date as the number of days from sowing to when 50% of the plants have shed the pollen, (ii) silking date as the number of days from sowing to when 50% of the plants have emerged the silk, (iii) pollen-shed duration as the number of days from the last day of pollen shedding minus the first day of pollen shedding, and (iv) pollen production by shaking the tassel at full-anthesis stage and visually scoring from 1 (excellent) to 5 (poor). At milk stage (R3), all families were measured on (i) plant height as the distance from ground level to the node bearing the flag leaf and (ii) ear height as the distance from ground level to the node bearing the uppermost ear. Both plant and ear heights were observed on ten plants per plot.

### 2.4. Visual Assessment of R1-nj Anthocyanin Expression on Ploidy Discrimination

**Figure 3:**
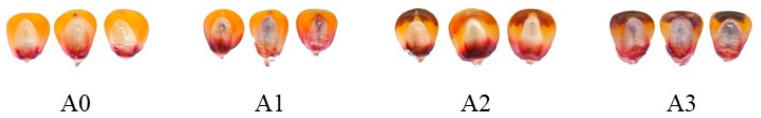
*R1-nj* anthocyanin marker expressed on the endosperm and embryo of dried tester seeds. From left to right: (**A0**) Out crossed or self-pollinated; (**A1**) Lethal; (**A2**) Putative haploid; (**A3**) Putative diploid.

Tester and inducer ears derived from haploid induction and line purification, respectively (Figure 2) were harvested at physiological maturity (R6) and dried under the sun for a few days to obtain the dried seeds with 11–12% of moisture content. Then, all seeds from each ear were classified based on *R1-nj* marker expression on the crown (top endosperm tissue) and scutellum of the embryo [31] into four groups: (i) A0, seeds without purple coloration of the endosperm and embryo, referred to as outcrossed or self-pollinated; (ii) A1, seeds with colorless endosperm and purple embryo; (iii) A2, seeds with purple endosperm and colorless embryo, referred to as putative haploid; (iv) A3, seeds with a purple coloration of the endosperm and embryo, referred to as putative diploid (Figure 3). Haploid induction rate (HIR) was calculated as the percentage of putative haploid seeds that can be generated per cross, whereas inducer seed rate (ISR) is the percentage of inducer seeds that can be maintained within an ear.

(1) $$\mathrm{HIR}\;(\%)=\frac{\mathrm{seed}\;\mathrm{number}\;\mathrm{of}\;\mathrm{putative}\;\mathrm{haploid}}{\mathrm{total}\;\mathrm{seed}\;\mathrm{number}\;\mathrm{per}\;\mathrm{ear}}\;\times \;100$$

(2) $$\mathrm{ISR}\;(\%)=\frac{\mathrm{seed}\;\mathrm{number}\;\mathrm{of}\;\mathrm{inducer}}{\mathrm{total}\;\mathrm{seed}\;\mathrm{number}\;\mathrm{per}\;\mathrm{ear}}\;\times \;100$$

**Figure 4:**
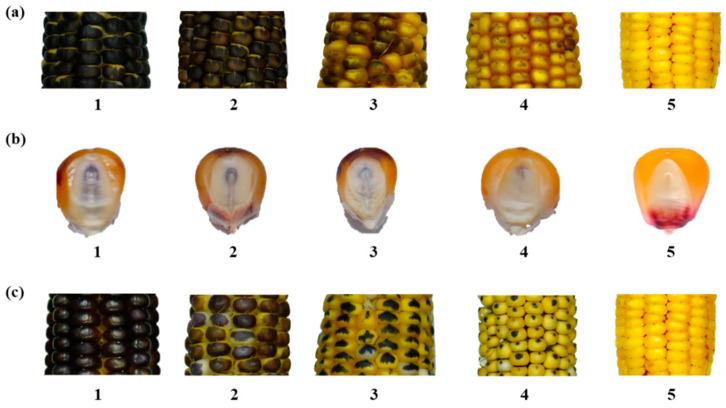
Variation in *R1-nj* anthocyanin expressions of dried seeds of putative haploid inducers. (**a**) Variation for intensity of purple coloration on the endosperm. (**b**) Variation for intensity of purple coloration on the embryo. (**c**) Variation for area marked of endosperm purple coloration.

Representative ten putative haploid inducer seeds of each family plot were used to visually score two parameters: (i) intensity of *R1-nj* marker pigmentation of endosperm (IED) and embryo (IEM) by using five rating scales from 1 (intense coloration) to 5 (no coloration) [32] and (ii) area marked of *R1-nj* marker pigmentation of endosperm (AED) by using five rating scales from 1 (almost full covering the entire aleurone layer of the endosperm) to 5 (completely lacking) [33] (Figure 4).

### 2.5. Statistical Analysis

Data for all observed traits derived from each breeding cycle (C1 to C3) was subjected to Bartlett’s test for homogeneity of variance and Shapiro–Wilk test for normality. A logarithmic transformation was performed on traits having non-normal distributions as:

y′ = log10 (y + 1)(3)

where y′ is the transformed data and y is the original data.

Then, combined analysis of variance (ANOVA) in RCBD was performed considering cycle as random effect and genotype as fixed effect by using PROC MIXED of SAS ver. 9.0 [34] using the following linear model:

Y_ijk_ = µ + c_i_ + r_j_(c_i_) + f_k_ + c_i_f_k_ + ε_ijk_ (4)

where i = 1, 2, 3; j = 1, 2; k = 1, 2, 3 … 78; Y_ijk_ denotes the phenotype of family k in cycle i and replication j; µ is the overall mean; c_i_ is the effect of cycle i; r_j_(c_i_) is the effect of replication k nested within cycle i; f_k_ is the effect of family k; c_i_f_k_ is the effect of the interaction between cycle i and family k; ε_ijk_ is the pooled error of cycle i, replication j, and family k.

Linear coefficient of regression (*b*) was calculated to determine the realized genetic gain per cycle on all observed traits over three breeding cycles [35]. Percentage increase due to selection (%Δ) was calculated as follows:

(5) $$\%\Delta =\frac{\left(y_{C3}-y_{C1}\right)}{y_{C1}}\;\times \;100\;$$

where $y_{C3}$ is phenotypic mean in breeding cycle 3 and $y_{C1}$ is phenotypic mean in breeding cycle 1.

Genetic parameters were estimated from the expected mean squares [36]. Broad-sense heritability was estimated using the variance ratio [37] as follows:

(6) $$h_{\mathrm{bs}}^{2}=\frac{\sigma_{g}^{2}}{\sigma_{g}^{2}{+(\sigma }_{\mathrm{gc}}^{2}{/c)+(\sigma }^{2}/\mathrm{rc})}\;$$

where $h_{\mathrm{bs}}^{2}$ is broad-sense heritability estimates; $\sigma_{g}^{2}$ is genotypic variance; $\sigma_{\mathrm{gc}}^{2}$ is variance of the interaction between cycle and genotype; $\sigma^{2}$ is variance of error; c is the number of breeding cycles; and r is the number of replications.

Genotypic (GCV) and phenotypic (PVC) coefficient of variation were calculated following Singh and Chaudhary [38] formula:

(7) $$\mathrm{GCV}\left(\%\right)=\frac{\sqrt{\sigma_{g}^{2}}}{\chi }\times \;100\;\;\;\mathrm{PCV}\left(\%\right)=\frac{\sqrt{\sigma_{p}^{2}}}{\chi }\times \;100$$

where $\sigma_{p}^{2}$ is phenotypic variance and χ is grand mean of the trait across breeding cycles.

Genetic advance (GA) for all observed traits was calculated following Singh and Chaudhary’s [38] formula:

(8) $${\mathrm{GA}=i\;\times \;\sigma }_{p}{\;\times \;h}_{\mathrm{bs}}^{2}$$

where i is selection intensity, which is 2.06 at 5%, and $\sigma_{p}$ is phenotypic standard deviation.

Genetic advance percentage (%GA) was calculated following Souza et al. [39] formula:

(9) $$\%\mathrm{GA}\;=\frac{\mathrm{GA}}{\chi }\;\times \;100$$

Dendrogram based on hierarchical Ward’s clustering method was constructed by JMP Pro software [40]. Duncan’s multiple range test (DMRT) at 0.05 probability level was used for mean comparison [35].

## 3. Results

### 3.1. Analysis of Variance

Cycle was significant for all observed traits (Table 1). Family was also significant for all observed traits. The interaction between cycle and family (C × F) was significant for anthesis date, silking date, ear height, haploid induction rate, haploid seed number per ear, inducer seed rate, and inducer seed number per ear. The significance of family indicated the phenotypic variation among 78 genotypes existed on agronomic traits and haploid induction ability. The significance of cycle indicated that all families showed different performances on agronomic traits and haploid induction ability across three breeding cycles; thus, genetic gains can be estimated. The significance of C × F on some traits suggested that there were different responses of each family to three breeding cycles. Based on the proportion of mean squares, cycle was consistently the major contributor on each observed trait, followed by family and C × F.

### 3.2. Realized Genetic Gain

**Figure 5:**
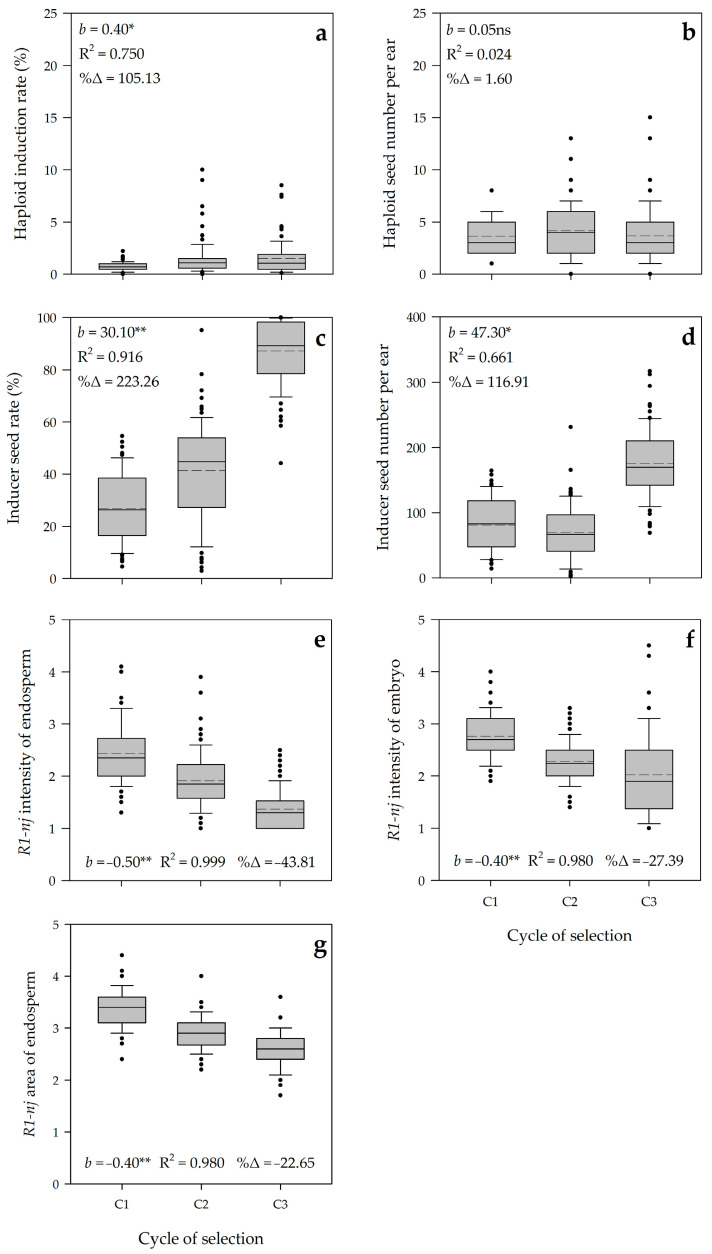
Parallel boxplots of 78 families of putative tropical haploid inducer in three cycles of selection for haploid induction ability. (**a**) Haploid induction rate (%); (**b**) Haploid seed number per ear; (**c**) Inducer seed rate (%); (**d**) Inducer seed number per ear (**e**) *R1-nj* intensity of endosperm; (**f**) *R1-nj* intensity of embryo; and (**g**) *R1-nj* area of endosperm. *b* realized genetic gain per cycle. R^2^ coefficient of determination. %Δ is percentage increase due to selection. *, ** *b* values are significantly different from zero at ≥2SE and ≥SE, respectively. ns *b* value is not significantly different from zero at ≥SE.

In general, the pattern of boxplots covering 78 families over three selection cycles was slightly increasing on haploid induction rate (HIR), but stagnant on haploid seed number per ear (HIE) (Figure 5). The significant increase was noticed on inducer seed rate (ISR) and inducer seed number per ear (ISE). In contrast, the selection trend was gradually decreasing on *R1-nj* intensity of endosperm (IED), *R1-nj* intensity of embryo (IEM), and *R1-nj* area of endosperm (AED).

**Figure 6:**
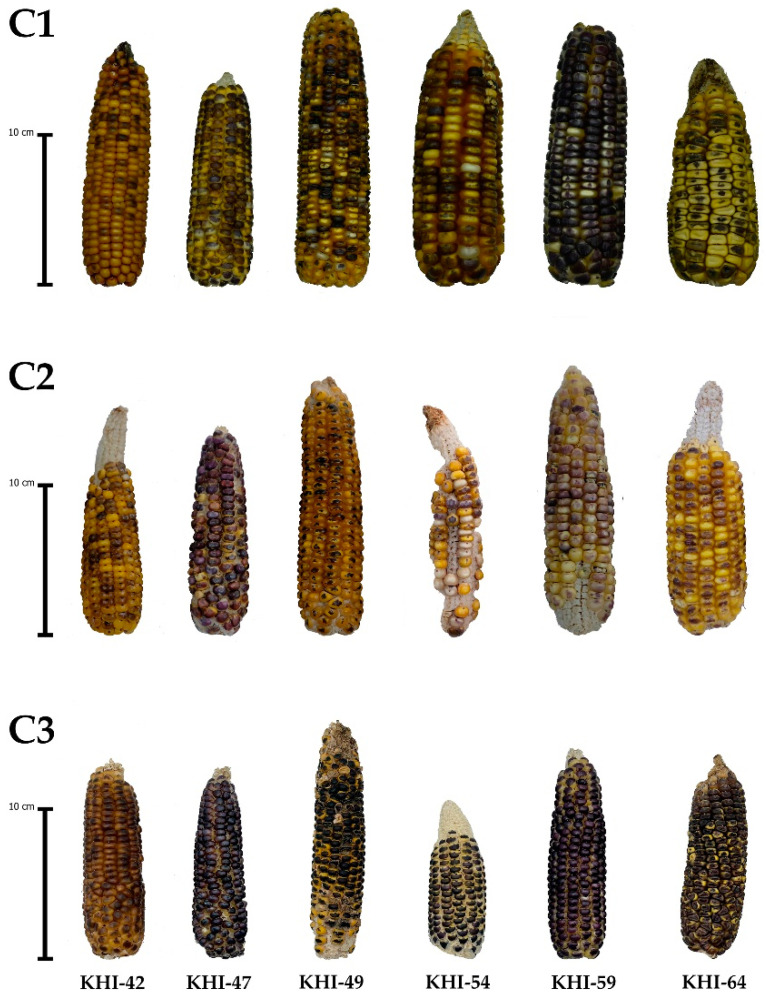
Representative corn ears of six families of putative tropical haploid inducer showing positive responses to three breeding cycles on inducer seed set and *R1-nj* anthocyanin expression (C1, C2, and C3 represent breeding cycle 1, cycle 2, and cycle 3).

The average realized genetic gains were positive and high for ISR (30.10% cycle^−1^) and ISE (47.30 seeds ear^−1^ cycle^−1^), representing substantial increases due to selection of 223.26% and 116.91%, respectively. The gain of selection was positive and low for HIR (0.4% cycle^−1^), but it was not significant and poor for HIE (0.05 seeds ear^−1^ cycle^−1^). On the contrary, the selection gain was negative and low for IED (−0.50 cycle^−1^), IEM (−0.40 cycle^−1^), and AED (−0.40 cycle^−1^), representing significant decreases due to selection of 43.81, 27.39, and 22.65%, respectively. This result indicated that modified ear-to-row selection applied in our population set was effective for enhancing the seed set of inducer families both their rate and seed number per ear. Besides this, negative gains of selection noticed on IED, IEM, and AED indicated that our selection method could improve the expression of *R1-nj* anthocyanin marker of inducer seeds (Figure 6). However, this selection method showed a low effectiveness to improve haploid induction rate.

The coefficient of determination (R^2^) for ISR, IED, IEM, and AED was high ranging from 0.916 to 0.999 while the values for HIR and ISE were moderate to high (R^2^ = 0.750 and 0.661, respectively). The high estimates of R^2^ indicated two things: phenotypic variation for those above traits was largely contributed by selection cycle, and the realized genetic gain was consistent in each cycle.

**Figure 7:**
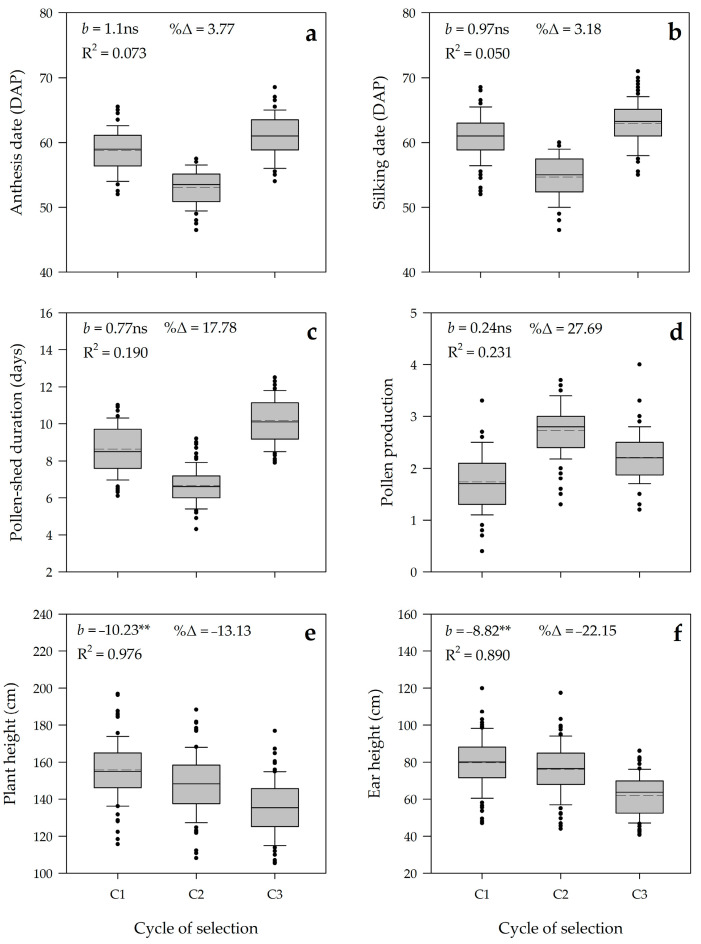
Parallel boxplots of 78 families of putative tropical haploid inducer for agronomic traits as an indirect response to three cycles of selection. (**a**) Anthesis date (DAP); (**b**) Silking date (DAP); (**c**) Pollen-shed duration (days); (**d**) Pollen production; (**e**) Plant height (cm); and (**f**) Ear height (cm). *b* realized genetic gain per cycle. R^2^ coefficient of determination. %Δ is percentage increase due to selection. ** *b* value is significantly different from zero at ≥2SE. ns *b* value is not significantly different from zero at ≥SE.

Meanwhile, for agronomic traits, the general pattern of boxplots covering 78 families over three selection cycles was fluctuating on anthesis date (DTA), silking date (DSI), pollen-shed duration (PSD), and pollen production (PPD) (Figure 7). The selection trend was slightly declining on plant height (PHE) and ear height (EHE). The average realized genetic gains were not significant for DTA (1.10 days cycle^−1^), DSI (0.97 days cycle^−1^), PSD (0.77 days cycle^−1^), and PPD (0.24 cycle^−1^), representing low percentage of increase due to selection variation from 3.18 to 27.69%. The gains of selection were negative and low for PHE (10.23 cm cycle^−1^) and EHE (8.82 cm cycle^−1^), representing minor decreases due to selection of 13.13% and 22.15%, respectively. The coefficient of determination (R^2^) for DTA, DSI, PSD, and PPD was low ranging from 0.073 to 0.231, while the values for PHE and EHE were high (R^2^ = 0.976 and 0.890, respectively). This result indicated that the fluctuating alterations of family means for flowering behaviors were probably contributed by seasonal variations during population improvements. Meanwhile, the slight decline of overall family means for plant and ear heights might be due to the inbreeding depression.

### 3.3. Genetic Parameters and Predicted Genetic Gain

Considerable genotypic variation was noticed on inducer seed rate (ISR), inducer seed number per ear (ISE), plant height (PHE), and ear height (EHE) varying from 299.73 to 3221.95 (Table 2). Meanwhile, other traits including haploid induction ability (HIR and HIE) had relatively low genotypic variation, ranging from 0.10 to 21.12. Heritability on ISR, ISE, DTA, DSI, PHE, and EHE were moderate to high ranging from 0.73 to 0.86, whereas the estimates on HIR and HIE were low to moderate accounting for 0.54 and 0.42, respectively. In general, genotypic coefficient of variation (GCV) on all traits varied from 7.34 to 70.05%, whereas phenotypic coefficient of variation (PCV) varied from 8.10 to 95.55%. The slight differences in which PCV was higher than GCV reflected the presence of environmental effects on all observed traits. Estimates of genetic advance (GA) were moderate on ISR (32.69) and high on ISE (105.86), representing 63.06 and 97.34% of GA, respectively. Haploid induction ability and the expression of *R1-nj* anthocyanin marker of inducer seeds showed relatively low GA estimates ranging from 0.38 to 1.92, representing 12.87 to 105.81% of GA.

### 3.4. Phenotypic Variation among Families

**Figure 8:**
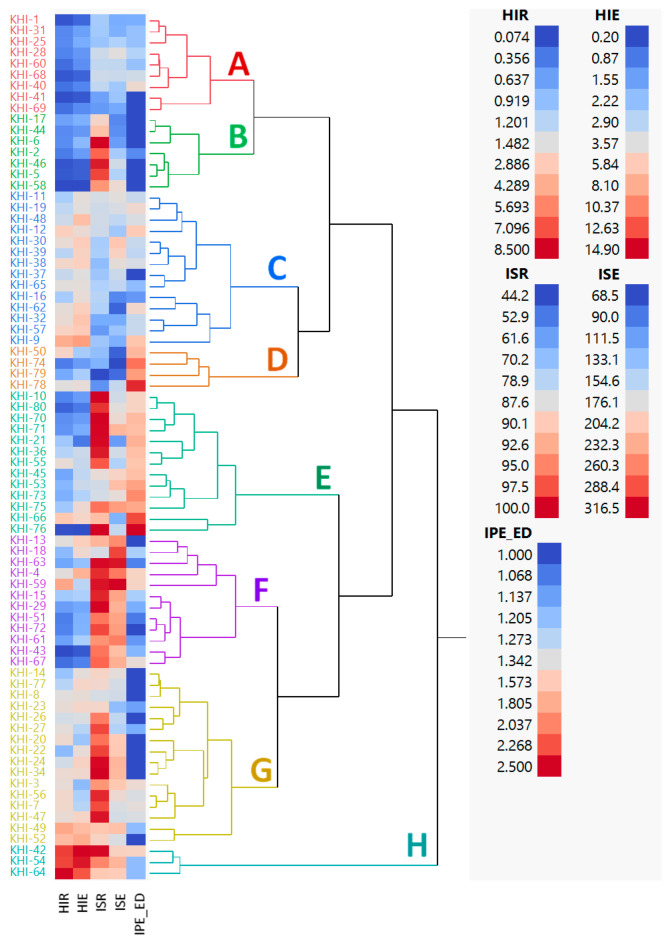
Dendrogram of phenotypic relationships among 78 families of putative tropical haploid inducer. Eight clusters (**A**–**H**) were formed. Hierarchical Ward’s clustering method was constructed based on haploid induction rate (HIR), haploid seed number per ear (HIE), inducer seed rate (ISR), inducer seed number per ear (ISE), and *R1-nj* intensity of endosperm (IPE_ED).

A dendrogram based on five selection criteria including haploid induction rate (HIR), haploid seed number per ear (HIE), inducer seed rate (ISR), inducer seed number per ear (ISE), and *R1-nj* intensity of endosperm (IED) classified 78 families into eight groups (A-H) (Figure 8). Group A was comprised of nine inducer families that had poor HIR (0.30%) and HIE (1.10 haploid seeds per ear), moderate ISR (74.67%) and ISE (143.46 inducer seeds per ear), and intense IED (1.20). Group B was comprised of seven inducer families with poor HIR (0.34%) and HIE (1.16 haploid seeds per ear), high ISR (95.47%), moderate ISE (137.12 inducer seeds per ear), and intense IED (1.01). Group C was comprised of 14 inducer families that had low HIR (1.86%), moderate HIE (5.23 haploid seeds per ear), ISR (75.85%), ISE (151.30 inducer seeds per ear), and IED (1.33). Group D was comprised of four inducer families with low HIR (1.24%) and HIE (2.68 haploid seeds per ear), moderate ISR (60.01%), low ISE (98.25 inducer seeds per ear), and moderate IED (2.05). Group E was comprised of 13 inducer families that had poor HIR (0.95%), low HIE (2.42 haploid seeds per ear), high ISR (95.37%), and moderate ISE (177.54 inducer seeds per ear) and IED (1.82). Group F was comprised of 12 inducer families that had low HIR (1.07%) and HIE (2.95 haploid seeds per ear), high ISR (96.25%) and ISE (258.43 inducer seeds per ear), and intense IED (1.21). Group G was comprised of 16 inducer families with low HIR (1.94%), moderate HIE (4.26 haploid seeds per ear), high ISR (94.39%), moderate ISE (183.81 inducer seeds per ear), and intense IED (1.12). Group H was comprised of three inducer families that had high HIR (7.82%), HIE (13.97 haploid seeds per ear), ISR (95.00%), ISE (208.06 inducer seeds per ear), and moderate IED (1.30). The dendrogram clearly distinguished three inducer families from the group H, namely, KHI-42, KHI-54, and KHI-64, that possessed good haploid induction ability, excellent inducer seed set, and moderate to intense *R1-nj* marker expression.

Phenotypic coefficient variation dynamics of a population set during three consecutive cycles of selection was declining for ISR, fluctuating for ISE, increasing for HIR and HIE, and stagnant for IEM and AED (Table 3). Meanwhile, on agronomic traits, the coefficient variation across cycles was stagnant for AD, SD, PH, and EH but slightly declining for PSD and PPD (Table 4). The result illustrated that consecutive cycles of self-pollination followed by high selection intensity (5%), according to the selection criteria, could narrow down the phenotypic variation among 78 families for inducer seed set but expand the phenotypic variation for haploid induction ability (HIR and HIE). Besides this, the intense selection based on *R1-nj* marker did not significantly interfere the phenotypic variation of 78 families on overall agronomic traits.

## 4. Discussion

### 4.1. Analysis of Variance

Three factors including family, cycle, and their interaction are prerequisites for crop population improvement programs because these reveal the first signal of significant genotypic variation of each family and their responses to selection method applied. In our study, all observed traits showed significant differences between both families and selection cycles. The effect of cycle and family interaction was significant only for some traits including haploid induction rate. This interaction effect reflected three directions of selection gains of each family including positive, negative, and no responses on respective traits. This variability in selection response can be due to genetic drift [41], confirming that conventional breeding through phenotypic selection is a numbers game [42].

Among sources of variation, cycle effect was predominant according to the mean square proportion on all parameters except on haploid seed number per ear. This might be contributed by the selection method chosen and the high selection intensity. This present study used 5% intensity of modified ear-to-row selection. Previous studies reported that cycle effect was also considerable when modified mass selection with 5–10% of selection intensity was performed on anthocyanin contents and their antioxidant activities in purple field corn [29] and on yield, yield components, and early maturity in purple waxy corn [43].

Coefficient of variation (*cv*) reflected the level of reliability of the experiment since it expressed the experimental error as mean percentage [35]. In our study, agronomic traits showed relatively low *cv*. However, a higher *cv* value was noticed on haploid induction rate (HIR). The *cv* value of this study was estimated from plot basis; thus, it indicated that variation of HIR among plants within a family was higher than that between families and HIR did not distribute normally. The frequency distribution on HIR of maize haploid inducer populations was reported to be right skewed [12,17,20,44]. Molecular evidence using SSR markers revealed the segregation distortion on a major locus *gg1* controlling in situ maternal haploid induction [13].

### 4.2. Genetic Parameters and Genetic Gains Reveal the Effectiveness of Modified Ear-to-Row with Intra-Family Selection

Population improvement over breeding cycles is essential to bring favorable alleles together [45]. Genetic gains and genetic parameters including heritability, phenotypic coefficient of variation (PCV), and genetic coefficient of variation (GCV) are commonly estimated to evaluate the breeding strategy applied on certain breeding objectives [46]. Genetic gain covers expected and realized genetic gains. Expected genetic gain is a predicted change in phenotype that would occur due to proposed breeding strategy while realized genetic gain is the observed change in phenotype due to selection over cycles [45]. For practical estimation, expected genetic gain on a particular trait is the product of its heritability, phenotypic standard deviation, and selection intensity [47] while realized genetic gain per cycle is derived from the slope of the linear regression of mean breeding value on certain cycle number [48].

Heritability reflects the phenotypic variation that is due to genetic effect and the estimates would be varied depending upon the genotypic differences within a population, environmental effect, and the interaction between genotype and environment [49]. In this study, genotypic variance and heritability estimate for inducer seed set were high ($\sigma_{g}^{2}$ = 299.73–3221.95; $h_{\mathrm{bs}}^{2}$ = 0.82–0.84) and might explain the considerable genetic gains for these traits. Thus, multi-phenotyping involving visual selection at harvest stage followed by counting inducer seed possessing *R1-nj* marker of both endosperm and embryo was effective. Considerable genetic variation existed within the population on a particular trait is required for realizing significant genetic gain [50], and high heritability is associated with high genetic gain [51]. Meanwhile, heritability estimate for HIR was moderate ($h_{\mathrm{bs}}^{2}$ = 0.54). This estimate was better than a previous study by Ribeiro et al. [52] that found low heritability on HIR ($h_{\mathrm{bs}}^{2}$ = 0.11–0.22). On the contrary, Lashermes and Beckert [20] reported a high heritability on HIR ($h_{\mathrm{bs}}^{2}$ = 0.93) using the parent–offspring regression. Likewise, Almeida et al. [53] applied genomic prediction for HIR and a high estimate ($h_{\mathrm{bs}}^{2}$ = 0.90) was noticed. Using QTL analysis, Prigge et al. [21] found moderate to high heritability estimates on HIR ($h_{\mathrm{bs}}^{2}$ = 0.32–0.80) derived from different biparental populations. In our study, moderate heritability on HIR was attributed by low genetic variance ($\sigma_{g}^{2}$ = 0.74). This low variation among families within a population set could be explained that all families derived from a common ancestor Stock6 as donor parent for haploid induction ability.

Current major objectives of breeding haploid inducer are improvements on haploid induction rate (HIR), agronomic performance, and adaptation to specific environments [6]. In this study, the target environment was regions under tropical savanna climate, and phenotypic selections were emphasized on agronomic performance including flowering behaviors and plant architecture, inducer seed set, and *R1-nj* marker expression of inducer seeds while HIR evaluation was monitored regularly. The breeding strategy was three cycles of modified ear-to-row with 5% of intra-family selection. All 78 families showed positive realized gains on inducer seed set while most families showed negative realized gains on *R1-nj* marker of endosperm and embryo (Table S2), indicating the effectivity of that strategy on inducer seed *R1-nj* expression and seed set. However, phenotypic selection applied showed a low effectiveness to improve haploid induction rate although some families had considerable HIR improvement. For instance, the HIR of family KHI-42 was increasing from 0.4 to 7.6% (Table S2). From 78 families, 12 families had significant positive gain, 27 families had negative gain, while the rest families showed not significant gain on HIR. Likewise, genetic advance showed the similar pattern with realized gain per cycle for each selection criterion.

The progress of breeding haploid inducers for advanced HIR have been reported in numerous studies (8–18,44,52,54), only a few of which could clearly explain the breeding strategy via phenotypic selection scheme. Aman and Sarkar [44] performed three cycles of full-sib with inter-family selection and could increase the HIR from 0.2% to above 3.0%. Rotarenco et al. [15] conducted initial crosses between two inducer lines (MHI and Stock6), performed phenotypic selections, and obtained four PHI families having high HIR (12.0–14.5%). Shatskaya [54] performed thirteen cycles of modified ear-to-row with combined individual and intra-family selections and could enhance the HIR from 0.1 to 13.1% in the ZMK inducer families. Later, Riberio et al. [52] utilized inducer line ZMK 1 to develop segregated families, performed family selections, and achieved the best family with HIR about 5.3%. They also noticed that intra-family selection resulted in higher genetic gains than inter-family selection, and increments of selection intensity from 50% to 10% could enhance the genetic gain on HIR. Prigge et al. [17] constructed the base populations from crosses between two inducer hybrids RWS × UH400 and RWS × RWK and three CMLs, performed mass selection on the F2 plants for agronomic performance and ear-to-row method on selected progenies for HIR, and obtained tropical inducer candidates having HIR up to 10%.

### 4.3. The Impact of Breeding Strategy on Family Distribution and Phenotypic Coefficient Variation (PVC) among Families

In this study, most families had low HIR ranging from 0.0 to 1.5%. However, hierarchical cluster analysis based on five selection criteria obviously noticed three inducer families KHI-42, KHI-54, and KHI-64 possessing HIR about 7.6%, 7.4%, and 8.5%, respectively (Table S2). These families also showed excellent inducer seed set and moderate to intense *R1-nj* marker expression. The HIR expressed in these families was immensely higher than other families and their ancestor, Stock6 (HIR = 2.3%) [7]. This reflected the presence of transgressive segregants after gene introgression for HIR performed [17,18,21]. Haploid induction ability is a complex trait influenced by many factors including environmental condition during haploid induction [12,55,56], inducer genetic [12,17], source germplasm [32,56,57], and the silk age of source germplasm [58]. Thus, the top three inducer families obtained in this study could be further evaluated on haploid induction ability and agronomic performance under multi-environment (year × season × location) and different source germplasm.

In this study, the dynamics of PVC reflected whether ear-to-row with intra-family selection altered the phenotypic variation among families in each breeding cycle (C1, C2, and C3). Significant reduction of PVC among families on inducer seed set from C1 to C3 indicated that the frequency of favorable alleles for *R1-nj* gene was well established in advanced breeding cycle. On the contrary, significant extension of PVC among families on haploid induction ability indicated the presences of genetic drift [41] and transgressive segregants for the HIR-linked alleles during selection. Meanwhile, our breeding strategy did not compromise the agronomic performance of inducer families, as indicated by low inbreeding depression and stable PVC across breeding cycles on plant height, ear height, anthesis date, silking date, pollen production, and pollen-shed duration. This result was preferable since one of major challenges hindering the adoption of temperate haploid inducer lines for the DH technology is that these genotypes exhibited poor tropical adaptation including poor pollen production, plant vigor, seed set, and susceptible to common tropical diseases [59]. Backcrossing method, an alternative breeding strategy by intercrossing between F1 progenies (50:50 inducer and non-inducer) and their adapted non-inducer parents, was reported to be effective for improving tropical adaptation of inducer candidates without sacrificing high HIR [17,18]. Previous investigations revealed the high heritability on agronomic traits and the weak trait associations between agronomic traits and HIR, indicating that recombining favorable alleles for HIR of temperate inducers and tropical adaptation is possible [17,53].

### 4.4. Future Breeding Strategies for Improving Genetic Gain on HIR

This study illustrated that phenotypic selection alone could realize positive but low genetic gain on HIR. The rates of realized gain are depending upon four factors: additive genetic variance, accuracy of selection, selection intensity, and duration of breeding cycle [45]. Expanding the genetic variance on HIR through additional cycles of introgression and evaluation is possible because HIR is a polygenic trait governed by several QTLs [21]. Thus, recombining all those favorable alleles might lead to the opportunity to obtain promising haploid inducers that surpass minimum threshold of HIR (10.0%) for practical use in the in vivo DH technology. Tightening the selection intensity could be implemented by increasing the population size and the scale of field trials [60]; however, in such breeding haploid inducer, phenotypic based HIR including in vivo haploid induction and visual discrimination of haploid and diploid seeds is costly, time consuming, and labor intensive [61]. Increasing the accuracy of selection on HIR through marker-assisted selection (MAS) [62] is more affordable since the genotyping cost is declining. The HIR-linked markers have extensively been studied and mapped through QTL analyses. Two major QTLs *qhir1* and *qhir8* located on chromosomes 1 and 9, respectively, were responsible for triggering HIR, and other several minor QTLs as complement were also identified [21]. Dong et al. [63] fine-mapped the *qhir1* locus to a 243 kb region flanked by markers X291 and X263 and so did Liu et al. [64] for the *qhir8* locus to a 789 kb region flanked by markers 4292232 and umc1867. Combining pedigree selection with MAS based on *qhir1* locus has been effective to fasten HIR improvement in high oil inducer lines [65] and the CIMMYT second-generation Tropically Adapted Inducer Lines (CIM2GTAILs) [18]. Besides this, Uliana Trentin et al. [61] suggested that breeding haploid inducer should be focused on at least four desirable alleles, namely, *R1-nj* and *Pl1* for ploidy marker system and *mtl* and *zmdmp* for HIR; however, the chance to fix these alleles altogether was low (~0.4%). Thus, they proposed MAS to fix the *mtl* allele in the F2 plants and the *zmdmp* allele in the F3 plants while fixation of *R1-nj* allele could be done by phenotypic selection. Those above reports indicated that implementing MAS for the HIR-favorable alleles in the early generation can reduce the number of F2 plants for further phenotyping stage, and only selected lines with acceptable levels of HIR (>10%) will be tested in the field. This would improve the genetic gain on HIR.

## 5. Conclusions

A positive but low selection gain was realized on haploid induction ability, whereas high desirable genetic gains were realized on inducer seed set and the *R1-nj* marker expression of both endosperm and embryo. Flowering behaviors, including flowering dates, pollen production, pollen-shed duration, had non-significant realized gain, while plant architecture such as plant and ear heights showed minor negative gains. All 78 families were clustered into eight groups, and three families KHI-42, KHI-54, and KHI-64 performed good haploid induction ability, excellent inducer seed set, and moderate to intense *R1-nj* marker expression. Stability and adaptability of these families on HIR and related important traits under different environments and source germplasm should be further investigated. Our breeding strategy involving introgression of exotic inducer Stock6 into tropical gene pool, initial modified mass selection, and further modified ear-to-row method with 5% intra-family selection was effective for improving inducer seed set and the *R1-nj* marker expression without compromising overall agronomic performance of inducer families. Further improvements on breeding strategy were suggested to enhance advanced genetic gain on HIR including additional cycles of recombination with elite, available haploid inducers, backcrossing method, and MAS approach.

## Figures and Tables

**Table 1:** Mean squares from the combined ANOVA for haploid induction ability and agronomic traits of 78 putative families across three breeding cycles.

Traits	Source of Variation				CV (%)
	Cycle (C)	Family (F)	C × F	Pooled Error	
df	2	77	154	231	
Agronomic traits					
Anthesis date	2647.4 **	39.7 **	9.9 **	3.9	3.4
Silking date	2805.5 **	49.2 **	10.5 **	5.0	3.7
Pollen-shed duration	480.2 **	4.3 **	1.8 ns	1.9	16.1
Pollen production	38.9 *	0.9 **	0.3 ns	0.4	22.6
Plant height	16,743.8 *	1153.1 **	188.1 ns	181.0	9.2
Ear height	13,651.3 *	853.4 **	81.2 *	63.2	10.9
Haploid induction ability					
Haploid induction rate ^1^	29.2 *	6.8 **	2.4 **	0.6	29.7
Haploid seed number per ear ^1^	9.8 *	19.7 **	7.3 **	2.8	24.4
Inducer seed rate	154,663.5 **	656.5 **	360.8 **	57.0	23.5
Inducer seed number per ear	528,600.6 **	7153.1 **	2593.8 **	709.2	24.5
*R1-nj* intensity of endosperm	44.1 *	1.0 **	0.3ns	0.3	18.1
*R1-nj* intensity of embryo	22.9 **	0.9 **	0.6ns	0.5	19.3
*R1-nj* area of endosperm	22.8 **	0.4 **	0.2ns	0.2	15.7

^1^ data was subjected to a logarithmic transformation. *, ** significant at *p* ≤ 0.05 and *p* ≤ 0.01, respectively. ns not significant.

**Table 2:** Estimates of genetic parameters and predicted genetic gain of haploid induction ability and agronomic traits at 5% intra-family selection.

Traits	$\sigma_{g}^{2}$	$\sigma_{p}^{2}$	$h_{bs}^{2}$	GCV (%)	PCV (%)	GA	%GA
Haploid induction ability							
Haploid induction rate	0.74	1.37	0.54	70.05	95.55	1.30	105.81
Haploid seed number per ear	2.05	4.85	0.42	38.00	58.44	1.92	50.90
Inducer seed rate	299.73	356.73	0.84	33.40	36.43	32.69	63.06
Inducer seed number per ear	3221.95	3931.15	0.82	52.19	57.65	105.86	97.34
*R1-nj* intensity of endosperm	0.33	0.62	0.54	30.24	41.28	0.87	45.63
*R1-nj* intensity of embryo	0.21	0.69	0.31	19.52	35.22	0.52	22.28
*R1-nj* area of endosperm	0.10	0.32	0.33	10.93	19.12	0.38	12.87
Agronomic traits							
Anthesis date	17.88	21.77	0.82	7.34	8.10	7.89	13.70
Silking date	21.12	26.13	0.81	7.70	8.57	8.51	14.27
Pollen-shed duration	1.19	3.04	0.39	12.84	20.55	1.40	16.53
Pollen production	0.26	0.64	0.41	23.00	35.91	0.68	30.36
Plant height	486.04	667.05	0.73	15.04	17.62	38.77	26.45
Ear height	395.07	458.28	0.86	27.36	29.47	38.02	52.33

$\sigma_{g}^{2}$ genotypic variance. $\sigma_{p}^{2}$ phenotypic variance. $h_{\mathrm{bs}}^{2}$ broad-sense heritability. GCV (%) genotypic coefficient of variation. PCV (%) phenotypic coefficient of variation. GA genetic advance. %GA genetic advance as a percentage of mean.

**Table 3:** Phenotypic coefficient variation (%) dynamics among families of putative tropical haploid inducer during three cycles of modified ear-to-row selection for haploid induction ability.

Cycle	ISR	ISE	HIR	HIE	IED	IEM	AED
C1	49.08 a	49.80 b	57.63 b	44.95 b	23.59 a	16.05 b	10.91 a
C2	45.51 a	60.99 a	118.09 a	61.18 b	29.31 a	17.14 b	12.03 a
C3	14.53 b	29.88 c	109.43 a	80.74 a	27.47 a	40.22 a	13.12 a

ISR, inducer seed rate. ISE, inducer seed number per ear. HIR, haploid induction rate. HIE, haploid seed number per ear. IED, *R1-nj* intensity of endosperm. IEM, *R1-nj* intensity of embryo. AED, *R1-nj* area of endosperm. Means with different letters in the same column are significantly different by Duncan’s multiple range test (DMRT) at 0.05 probability level.

**Table 4:** Phenotypic coefficient variation (%) dynamics among families of putative tropical haploid inducer during three cycles of modified ear-to-row selection for agronomic traits.

Cycle	AD	SD	PSD	PPD	PH	EH
C1	5.73 a	5.81 a	14.80 a	30.70 a	10.38 a	17.24 a
C2	5.22 a	5.93 a	14.55 a	17.10 b	11.11 a	18.08 a
C3	5.35 a	5.45 a	11.47 b	22.01 b	11.22 a	18.32 a

AD anthesis date. SD silking date. PSD pollen-shed duration. PPD pollen production. PH plant height. EH ear height. Means with different letters in the same column are significantly different by Duncan’s multiple range test (DMRT) at 0.05 probability level.

## Data Availability

The data that support the findings of this study are available from the corresponding author upon reasonable request.

## Supplementary Materials

The following are available online at www.mdpi.com/article/10.3390/plants10122812/s1, Table S1: list of 78 families of putative haploid inducers used in this study, Table S2: means for haploid seed number per ear (HSE), haploid induction rate (HIR), inducer seed number per ear (ISE), inducer seed rate (ISR), *R1-nj* intensity of endosperm (IED), *R1-nj* intensity of embryo (IEM), and *R1-nj* area of endosperm (AED) during three cycles of modified ear-to-row selection.
