# Supplementary material for: Selection Gain of Maize Haploid Inducers for the Tropical Savanna Environments
Source: Plants (Basel). 2021 Dec 19;10(12):2812. doi: 10.3390/plants10122812 (PMC8706992; doi:10.3390/plants10122812)
Supplement: Supplementary file 1 [file plants-10-02812-s001.zip › plants-1520855-supplementary.pdf]

Supplementary Table

Supplementary Table S1. List of 78 families of putative haploid inducers used in this study.

No	Family code	Pedigree
1	KHI1	TIL1A-100-2-4
2	KHI2	TIL1A-105-B-B5
3	KHI3	TIL1A-108-B-18
4	KHI4	TIL1A-10-B-1
5	KHI5	TIL1A-110-B-11
6	KHI6	TIL1A-20-B-7
7	KHI7	TIL1A-21-B-4
8	KHI8	TIL1A-22-B-B2
9	KHI9	TIL1A-26-B-B2
10	KHI10	TIL1A-28-B-B2
11	KHI11	TIL1A-29-B-B2
12	KHI12	TIL1A-2-B-B2
13	KHI13	TIL1A-31-B-6
14	KHI14	TIL1A-36-B-B2
15	KHI15	TIL1A-37-B-2
16	KHI16	TIL1A-3-B-B2
17	KHI17	TIL1A-41-B-B4
18	KHI18	TIL1A-43-B-7
19	KHI19	TIL1A-45-B-4
20	KHI20	TIL1A-56-B-8
21	KHI21	TIL1A-57-B-2
22	KHI22	TIL1A-5-B-5
23	KHI23	TIL1A-70-B-6
24	KHI24	TIL1A-71-B-6
25	KHI25	TIL1A-80-B-12
26	KHI26	TIL1A-92-B-15
27	KHI27	TIL1A-93-B-4
28	KHI28	TIL1A-WS-B-B3
29	KHI29	TIL1B-59-B-1
30	KHI30	TIL1B-62-B-9
31	KHI31	TIL1B-65-1-B2
32	KHI32	TIL1B-72-B-2
33	KHI34	TIL1B-75-B-17
34	KHI36	TIL1B-78-B-2
35	KHI37	TIL1B-79-B-B2
36	KHI38	TIL1B-81-3-1
37	KHI39	TIL1B-84-B-2
38	KHI40	TIL1B-85-B-B3
39	KHI41	TIL1B-89-B-1

No	Family code	Pedigree
40	KHI42	TIL1B-93-B-9
41	KHI43	TIL2A-16-B-2
42	KHI44	TIL2A-17-B-1
43	KHI45	TIL2A-18-B-24
44	KHI46	TIL2A-21-B-2
45	KHI47	TIL2A-24-B-13
46	KHI48	TIL2A-27-B-B2
47	KHI49	TIL2A-28-B-8
48	KHI50	TIL2A-29-B-8
49	KHI51	TIL2A-30-B-11
50	KHI52	TIL2A-32-B-B8
51	KHI53	TIL2A-33-B-B2
52	KHI54	TIL2A-34-1-14
53	KHI55	TIL2A-35-B-10
54	KHI56	TIL2A-36-B-5
55	KHI57	TIL2A-37-B-4
56	KHI58	TIL2A-7-B-2
57	KHI59	TIL2A-WS-B-7
58	KHI60	TIL2B-13-1-B2
59	KHI61	TIL2B-15-B-3
60	KHI62	TIL2B-16-1-B3
61	KHI63	TIL2B-18-B-4
62	KHI64	TIL2B-21-B-9
63	KHI65	TIL2B-22-B-4
64	KHI66	TIL3-4-B-1
65	KHI67	TIL4-10-B-8
66	KHI68	TIL4-12-B-B3
67	KHI69	TIL4-15-1-B2
68	KHI70	TIL4-18-B-1
69	KHI71	TIL4-22-B-4
70	KHI72	TIL4-24-B-12
71	KHI73	TIL4-26-B-B2
72	KHI74	TIL4-27-B-B2
73	KHI75	TIL4-28-B-B2
74	KHI76	TIL4-30-B-2
75	KHI77	TIL4-31-B-B3
76	KHI78	TIL4-34-B-2
77	KHI79	TIL4-3-B-B
78	KHI80	TIL4-4-B-B2

Supplementary Table S2. Means for haploid seed number per ear (HSE), haploid induction rate (HIR), inducer seed number per ear (ISE), inducer seed rate (ISR), *R1-nj* intensity of endosperm (IED), *R1-nj* intensity of embryo (IEM), and *R1-nj* area of endosperm (AED) during three cycles of modified ear-to-row selection.

entry	family	cycle	ISR	ISE	HIR	HIE	IED	IEM	AED
1	KHI1	C1	35.4	131.0	0.3	1.5	1.9	2.8	3.1
		C2	43.3	90.1	0.9	3.8	1.5	2.5	3.0
		C3	74.4	119.6	0.1	0.6	1.2	2.0	2.5
		ΔC	39.0	-11.4	-0.1	-0.9	-0.8	-0.8	-0.6
		%ΔC	110.2	-8.7	-45.5	-60.0	-40.0	-29.2	-18.1
		b	19.5	-5.7	-0.1	-0.5	-0.4	-0.4	-0.3
		R^2	0.894	0.074	0.017	0.077	0.993	0.985	0.821
2	KHI2	C1	17.2	71.0	0.6	4.5	3.0	2.7	3.5
		C2	17.9	8.8	0.9	2.5	2.7	2.2	2.8
		C3	96.7	144.5	0.4	1.5	1.1	2.3	2.6
		ΔC	79.4	73.5	-0.2	-3.0	-1.9	-0.4	-0.9
		%ΔC	460.6	103.5	-38.5	-66.7	-63.0	-15.5	-25.3
		b	39.7	36.8	-0.1	-1.5	-0.9	-0.2	-0.4
		R^2	0.756	0.300	0.246	0.964	0.845	0.612	0.891
3	KHI3	C1	46.0	142.3	1.0	5.0	1.9	2.6	3.0
		C2	7.9	14.0	1.4	2.7	1.3	2.2	2.9
		C3	94.0	208.2	1.9	2.2	1.4	1.5	2.4
		ΔC	48.0	65.9	0.9	-2.8	-0.5	-1.1	-0.6
		%ΔC	104.2	46.3	94.5	-56.7	-24.4	-43.3	-19.1
		b	24.0	33.0	0.5	-1.4	-0.2	-0.6	-0.3
		R^2	0.308	0.111	0.999	0.878	0.565	0.950	0.930
4	KHI4	C1	38.3	118.7	1.1	5.3	3.3	3.0	3.1
		C2	43.2	43.4	1.5	5.1	2.6	2.2	2.9
		C3	98.9	266.2	1.6	7.7	1.5	2.0	2.9
		ΔC	60.6	147.5	0.5	2.5	-1.8	-1.0	-0.2
		%ΔC	158.0	124.3	50.6	46.7	-54.0	-34.8	-5.9
		b	30.3	73.8	0.3	1.2	-0.9	-0.5	-0.1
		R^2	0.810	0.424	0.937	0.712	0.971	0.910	0.750
5	KHI5	C1	29.5	114.3	0.1	1.0	2.3	2.8	3.4
		C2	49.3	101.7	0.6	2.4	1.8	2.1	3.0
		C3	98.0	151.0	0.2	0.5	1.0	2.5	2.6
		ΔC	68.5	36.7	0.0	-0.5	-1.3	-0.3	-0.8
		%ΔC	232.2	32.1	20.0	-50.0	-56.9	-12.4	-23.4
		b	34.3	18.3	0.0	-0.3	-0.7	-0.2	-0.4
		R^2	0.944	0.512	0.003	0.063	0.988	0.238	0.999
6	KHI6	C1	10.8	44.8	0.6	2.8	3.3	2.1	3.8
		C2	49.1	58.4	0.9	5.9	2.8	1.4	3.5
		C3	100.0	110.3	0.5	2.1	1.0	1.9	2.1

		ΔC	89.2	65.5	-0.1	-0.7	-2.3	-0.3	-1.7
		%ΔC	828.9	146.4	-11.8	-23.6	-69.8	-12.7	-45.1
		b	44.6	32.8	0.0	-0.3	-1.2	-0.1	-0.9
		R^2	0.993	0.898	0.031	0.026	0.897	0.135	0.886
7	KHI7	C1	32.2	95.8	0.3	1.8	3.1	2.7	3.3
		C2	55.6	111.7	0.4	2.9	2.5	2.2	2.6
		C3	97.8	173.3	2.1	2.9	1.4	1.9	2.4
		ΔC	65.7	77.4	1.8	1.2	-1.7	-0.9	-0.9
		%ΔC	204.1	80.8	608.3	65.7	-55.1	-31.3	-27.9
		b	32.8	38.7	0.9	0.6	-0.8	-0.4	-0.5
		R^2	0.974	0.896	0.796	0.766	0.975	0.989	0.904
8	KHI8	C1	30.7	74.0	0.6	3.8	2.0	2.8	3.8
		C2	17.9	35.3	0.9	2.6	1.2	2.3	3.1
		C3	85.6	168.0	1.6	4.1	1.0	1.0	2.3
		ΔC	54.9	94.0	1.0	0.3	-1.0	-1.8	-1.4
		%ΔC	179.1	127.0	174.7	8.9	-49.6	-64.5	-38.4
		b	27.5	47.0	0.5	0.2	-0.5	-0.9	-0.7
		R^2	0.583	0.474	0.979	0.048	0.909	0.931	0.997
9	KHI9	C1	41.0	117.5	0.5	3.5	2.7	2.5	3.0
		C2	57.1	112.8	1.1	3.5	2.0	2.1	2.6
		C3	73.1	122.3	4.3	9.0	1.6	1.3	2.1
		ΔC	32.1	4.8	3.8	5.5	-1.1	-1.2	-0.9
		%ΔC	78.2	4.1	731.9	157.1	-39.7	-49.3	-29.7
		b	16.0	2.4	1.9	2.8	-0.5	-0.6	-0.4
		R^2	0.999	0.257	0.868	0.750	0.985	0.949	0.999
15	KHI15	C1	25.4	73.0	1.0	3.8	2.9	3.6	3.3
		C2	46.3	109.2	1.6	7.2	2.5	2.8	2.4
		C3	99.2	237.8	1.0	2.7	1.3	2.8	2.8
		ΔC	73.8	164.8	0.0	-1.1	-1.6	-0.8	-0.5
		%ΔC	290.7	225.8	0.7	-29.6	-56.3	-21.5	-14.6
		b	36.9	82.4	0.0	-0.6	-0.8	-0.4	-0.2
		R^2	0.941	0.905	0.001	0.060	0.930	0.758	0.299
16	KHI16	C1	26.1	99.0	0.7	2.0	2.2	2.3	2.7
		C2	49.5	39.4	0.9	4.2	1.8	1.9	2.2
		C3	78.6	97.6	1.1	4.1	1.1	1.1	2.5
		ΔC	52.5	-1.4	0.4	2.1	-1.1	-1.1	-0.2
		%ΔC	200.8	-1.4	49.1	105.8	-48.8	-50.3	-7.6
		b	26.2	-0.7	0.2	1.1	-0.5	-0.6	-0.1
		R^2	0.996	0.001	0.981	0.710	0.966	0.948	0.149
17	KHI17	C1	13.7	44.2	0.7	4.0	2.5	2.9	3.6
		C2	21.3	20.2	1.3	4.4	1.9	2.3	2.8
		C3	89.1	98.4	0.7	1.9	1.0	1.4	2.8
		ΔC	75.4	54.2	0.0	-2.1	-1.5	-1.5	-0.7
		%ΔC	549.5	122.6	-5.2	-52.9	-59.5	-50.7	-20.8

		b	37.7	27.1	-0.1	1.1	-0.7	-0.7	-0.4
		R^2	0.825	0.457	0.001	0.710	0.989	0.993	0.760
18	KHI18	C1	13.1	35.1	0.5	3.1	3.0	2.3	2.9
		C2	50.0	125.1	0.8	7.4	2.1	1.9	2.5
		C3	85.4	294.4	0.9	4.5	1.3	1.0	2.9
		ΔC	72.3	259.3	0.4	1.4	-1.7	-1.3	0.0
		%ΔC	552.0	739.4	69.3	44.0	-57.8	-57.2	-1.0
		b	36.2	129.6	0.2	0.7	-0.9	-0.7	0.0
		R^2	0.999	0.970	0.924	0.102	0.999	0.976	0.003
19	KHI19	C1	21.1	60.3	1.2	5.0	3.1	3.3	3.7
		C2	24.1	37.6	1.7	3.9	2.3	2.6	3.1
		C3	83.8	172.4	1.3	3.7	1.4	1.5	2.2
		ΔC	62.7	112.2	0.0	-1.3	-1.6	-1.7	-1.5
		%ΔC	296.9	186.2	0.8	-26.0	-53.2	-53.6	-40.1
		b	31.4	56.1	0.1	-0.7	-0.8	-0.9	-0.7
		R^2	0.786	0.603	0.001	0.849	0.998	0.986	0.985
20	KHI20	C1	40.1	130.3	0.2	2.8	2.6	3.3	3.5
		C2	56.6	127.4	0.5	3.1	2.1	2.8	3.1
		C3	96.0	204.5	2.0	2.4	1.0	2.8	2.8
		ΔC	55.9	74.1	1.8	-0.3	-1.6	-0.5	-0.7
		%ΔC	139.6	56.9	908.2	-12.1	-61.3	-15.8	-20.9
		b	28.0	37.1	0.9	-0.2	-0.8	-0.3	-0.4
		R^2	0.947	0.721	0.860	0.221	0.944	0.750	0.993
21	KHI21	C1	27.0	91.6	1.2	4.5	1.8	2.8	3.6
		C2	50.0	86.4	1.8	6.6	1.2	2.4	3.1
		C3	99.8	110.1	1.0	0.9	1.8	1.2	2.4
		ΔC	72.8	18.5	-0.2	-3.6	0.0	-1.6	-1.1
		%ΔC	269.6	20.2	-13.1	-80.6	-0.2	-57.8	-32.0
		b	36.4	9.3	-0.1	-1.8	0.0	-0.8	-0.6
		R^2	0.957	0.554	0.042	0.398	0.001	0.924	0.999
22	KHI22	C1	19.7	52.3	2.2	7.8	2.1	3.8	3.2
		C2	30.2	48.3	2.8	8.1	1.6	3.2	2.9
		C3	98.1	206.8	0.9	4.0	1.0	1.2	2.8
		ΔC	78.4	154.6	-1.3	-3.8	-1.1	-2.7	-0.4
		%ΔC	397.2	295.7	-60.2	-48.4	-51.7	-69.5	-11.6
		b	39.2	77.3	-0.7	-1.9	-0.5	-1.3	-0.2
		R^2	0.848	0.731	0.462	0.676	0.993	0.912	0.860
23	KHI23	C1	41.5	123.8	0.4	3.8	2.4	3.1	3.5
		C2	54.5	76.2	0.9	3.6	1.9	2.7	3.3
		C3	89.0	132.2	1.8	4.8	1.1	1.0	2.3
		ΔC	47.4	8.4	1.4	1.1	-1.3	-2.1	-1.3
		%ΔC	114.2	6.8	405.5	28.0	-52.8	-67.9	-36.2
		b	23.7	4.2	0.7	0.5	-0.6	-1.1	-0.6
		R^2	0.935	0.020	0.974	0.662	0.989	0.899	0.908

24	KHI24	C1	17.0	33.6	0.8	3.3	3.1	2.5	3.0
		C2	42.8	62.6	1.1	4.3	2.6	2.3	2.7
		C3	99.8	223.4	1.4	5.2	1.0	1.7	2.8
		ΔC	82.8	189.8	0.5	1.9	-2.1	-0.8	-0.2
		%ΔC	485.6	564.9	65.3	58.5	-67.7	-32.9	-6.9
		b	41.4	95.0	0.3	1.0	-1.0	-0.4	-0.1
		R^2	0.955	0.862	0.999	0.995	0.920	0.956	0.452
25	KHI25	C1	47.5	143.9	0.6	2.5	2.6	3.3	3.4
		C2	48.1	93.9	1.2	4.8	2.0	2.5	2.9
		C3	72.3	149.9	0.5	1.8	1.2	1.3	2.8
		ΔC	24.8	6.0	-0.1	-0.7	-1.3	-2.0	-0.6
		%ΔC	52.2	4.2	-20.3	-28.0	-52.4	-60.0	-17.8
		b	12.4	3.0	-0.1	-0.4	-0.7	-1.0	-0.3
		R^2	0.768	0.010	0.022	0.052	0.982	0.987	0.865
26	KHI26	C1	27.6	86.8	1.0	3.3	2.8	3.1	3.0
		C2	33.6	64.4	1.7	4.6	2.3	2.4	2.6
		C3	95.4	159.3	1.4	3.5	1.0	1.9	2.0
		ΔC	67.8	72.6	0.4	0.3	-1.8	-1.1	-1.0
		%ΔC	246.1	83.6	34.6	7.7	-64.8	-37.3	-32.0
		b	33.9	36.3	0.2	0.1	-0.9	-0.6	-0.5
		R^2	0.816	0.535	0.271	0.029	0.937	0.989	0.993
27	KHI27	C1	7.4	28.0	0.9	4.3	1.5	2.5	3.3
		C2	34.9	55.2	1.3	4.6	1.1	2.2	2.6
		C3	98.0	151.3	1.7	2.9	1.2	2.4	2.8
		ΔC	90.6	123.3	0.8	-1.4	-0.4	0.0	-0.5
		%ΔC	1222.8	440.5	83.0	-31.8	-24.3	-2.0	-14.9
		b	45.3	61.7	0.4	-0.7	-0.2	-0.1	-0.2
		R^2	0.951	0.906	0.999	0.553	0.568	0.026	0.429
28	KHI28	C1	42.5	66.0	0.0	1.5	2.3	2.8	3.4
		C2	49.0	59.3	0.1	0.5	1.9	2.5	3.1
		C3	83.3	177.8	0.4	1.4	1.3	2.7	2.7
		ΔC	40.8	111.8	0.4	-0.1	-1.0	-0.1	-0.7
		%ΔC	95.9	169.3	1261.5	-6.7	-44.6	-3.1	-19.9
		b	20.4	55.9	0.2	-0.1	-0.5	-0.1	-0.3
		R^2	0.865	0.705	0.838	0.008	0.993	0.111	0.988
29	KHI29	C1	10.9	39.5	0.5	2.8	2.2	2.2	3.1
		C2	32.5	63.0	1.2	7.1	1.7	2.0	2.7
		C3	99.9	227.7	0.6	1.7	1.2	1.7	2.6
		ΔC	89.0	188.2	0.1	-1.1	-1.0	-0.5	-0.5
		%ΔC	816.2	476.4	15.9	-38.2	-46.9	-23.9	-17.1
		b	44.5	94.1	0.1	-0.5	-0.5	-0.3	-0.3
		R^2	0.919	0.842	0.012	0.033	0.999	0.986	0.941
30	KHI30	C1	21.1	86.1	0.7	2.0	2.2	3.1	3.6
		C2	43.8	69.8	1.3	6.5	1.7	2.4	2.9

		C3	75.3	208.5	1.9	5.7	1.3	1.4	2.5
		ΔC	54.1	122.4	1.1	3.7	-0.9	-1.7	-1.1
		%ΔC	256.2	142.0	161.1	185.0	-41.1	-55.0	-30.3
		b	27.1	61.2	0.6	1.9	-0.4	-0.9	-0.5
		R^2	0.991	0.651	0.999	0.594	0.999	0.984	0.968
31	KHI31	C1	28.1	65.5	0.8	5.5	1.7	3.3	3.7
		C2	30.8	18.0	1.0	5.7	1.3	2.8	3.1
		C3	74.3	122.0	0.5	1.6	1.2	1.3	2.6
		ΔC	46.2	56.5	-0.3	-3.9	-0.5	-2.0	-1.0
		%ΔC	164.6	86.3	-42.2	-71.4	-27.9	-59.7	-28.2
		b	23.1	28.3	-0.2	-1.9	-0.2	-1.0	-0.5
		R^2	0.795	0.294	0.398	0.714	0.807	0.931	0.988
32	KHI32	C1	18.5	55.2	1.1	4.5	2.7	2.7	3.3
		C2	35.4	96.0	1.8	5.6	1.8	2.3	2.8
		C3	64.6	118.4	1.9	6.3	1.3	1.7	2.5
		ΔC	46.1	63.2	0.8	1.8	-1.4	-1.0	-0.8
		%ΔC	248.7	114.4	71.1	39.7	-52.5	-37.1	-24.7
		b	23.0	31.6	0.4	0.9	-0.7	-0.5	-0.4
		R^2	0.977	0.973	0.871	0.976	0.970	0.995	0.960
34	KHI34	C1	30.7	97.0	0.2	2.5	2.5	3.2	4.1
		C2	63.5	93.5	0.3	3.8	2.3	2.8	3.5
		C3	100.0	221.2	1.9	4.6	1.0	3.1	2.6
		ΔC	69.3	124.2	1.7	2.1	-1.5	-0.1	-1.4
		%ΔC	225.2	128.0	863.7	84.0	-60.3	-3.1	-35.2
		b	34.6	62.1	0.9	1.1	-0.8	-0.1	-0.7
		R^2	0.999	0.729	0.782	0.988	0.860	0.061	0.979
36	KHI36	C1	19.7	64.9	0.7	3.8	2.7	2.5	3.5
		C2	17.9	29.3	1.1	6.0	2.2	2.3	2.8
		C3	99.5	169.2	1.2	3.2	1.7	2.1	2.6
		ΔC	79.7	104.3	0.4	-0.6	-1.1	-0.4	-0.9
		%ΔC	403.7	160.7	61.4	-14.7	-39.4	-16.5	-25.9
		b	39.8	52.1	0.2	-0.3	-0.5	-0.2	-0.5
		R^2	0.733	0.514	0.836	0.035	0.999	0.999	0.879
37	KHI37	C1	45.2	164.3	0.9	5.0	3.2	2.7	3.5
		C2	27.0	41.4	1.3	6.3	2.2	2.4	2.5
		C3	72.9	164.8	1.3	3.7	1.0	1.0	2.9
		ΔC	27.7	0.6	0.4	-1.4	-2.2	-1.7	-0.6
		%ΔC	61.2	0.3	39.0	-27.0	-68.3	-63.3	-16.7
		b	13.8	0.3	0.2	-0.7	-1.1	-0.9	-0.3
		R^2	0.358	0.001	0.831	0.270	0.995	0.901	0.366
38	KHI38	C1	37.6	85.8	0.5	3.0	1.9	3.3	3.4
		C2	24.6	44.1	1.5	6.0	1.6	2.6	2.9
		C3	69.8	181.2	2.2	5.4	1.3	1.1	2.5
		ΔC	32.2	95.4	1.7	2.4	-0.6	-2.2	-0.9

		%ΔC	85.7	111.3	354.6	80.0	-29.4	-67.1	-27.5
		b	16.1	47.7	0.9	1.2	-0.3	-1.1	-0.5
		R^2	0.479	0.461	0.995	0.571	0.997	0.956	0.999
39	KHI39	C1	28.0	85.8	1.7	7.5	2.5	3.2	3.5
		C2	47.7	95.4	2.1	5.9	1.7	2.5	3.2
		C3	75.9	196.9	1.2	5.2	1.3	1.4	2.4
		ΔC	47.9	111.2	-0.5	-2.4	-1.2	-1.8	-1.1
		%ΔC	170.8	129.6	-30.5	-31.3	-48.5	-56.9	-30.8
		b	24.0	55.6	-0.3	-1.2	-0.6	-0.9	-0.5
		R^2	0.989	0.814	0.383	0.957	0.979	0.981	0.957
40	KHI40	C1	10.4	21.0	0.6	3.8	1.8	2.7	3.4
		C2	47.2	47.4	1.3	5.4	1.4	1.9	2.8
		C3	80.7	141.6	0.3	1.1	1.4	1.9	2.2
		ΔC	70.4	120.6	-0.2	-2.7	-0.3	-0.8	-1.2
		%ΔC	678.6	574.2	-43.8	-71.1	-18.5	-28.2	-34.5
		b	35.2	60.3	-0.1	-1.3	-0.2	-0.4	-0.6
		R^2	0.999	0.905	0.064	0.375	0.644	0.699	0.999
41	KHI41	C1	19.5	53.3	0.5	2.0	3.3	2.4	3.0
		C2	51.1	132.1	0.9	3.8	2.9	1.8	2.4
		C3	62.0	139.4	0.1	0.4	1.0	2.5	1.9
		ΔC	42.5	86.1	-0.4	-1.6	-2.3	0.0	-1.1
		%ΔC	217.2	161.6	-78.5	-80.0	-69.9	0.3	-36.2
		b	21.2	43.1	-0.2	-0.8	-1.2	0.0	-0.6
		R^2	0.927	0.814	0.235	0.228	0.887	0.001	0.991
42	KHI42	C1	6.8	31.3	0.4	4.0	2.3	2.0	2.4
		C2	40.3	116.0	6.5	11.5	1.6	1.6	2.3
		C3	99.8	197.3	7.6	14.9	1.5	2.2	2.2
		ΔC	93.0	166.0	7.2	10.9	-0.8	0.2	-0.2
		%ΔC	1372.4	530.2	2038.2	272.5	-34.2	9.1	-8.1
		b	46.5	83.0	3.6	5.5	-0.4	0.1	-0.1
		R^2	0.975	0.999	0.860	0.957	0.805	0.103	0.997
43	KHI43	C1	50.5	130.2	0.7	2.3	2.6	3.4	3.1
		C2	51.4	60.2	0.0	0.0	1.8	3.0	2.9
		C3	95.8	216.6	0.1	0.4	1.2	2.3	2.1
		ΔC	45.4	86.4	-0.7	-1.9	-1.4	-1.1	-1.0
		%ΔC	89.9	66.3	-90.1	-82.2	-54.2	-32.6	-33.0
		b	22.7	43.2	-0.3	-0.9	-0.7	-0.6	-0.5
		R^2	0.765	0.304	0.669	0.594	0.997	0.970	0.924
44	KHI44	C1	10.8	13.8	1.2	4.2	3.0	2.5	3.4
		C2	4.2	3.8	0.3	2.1	2.1	2.2	3.1
		C3	91.0	102.5	0.5	1.4	1.0	3.1	3.0
		ΔC	80.2	88.7	-0.8	-2.7	-2.0	0.6	-0.4
		%ΔC	745.1	641.0	-60.9	-65.7	-66.5	23.5	-12.1
		b	40.1	44.3	-0.4	-1.4	-0.9	0.3	-0.2

		R^2	0.689	0.666	0.533	0.929	0.998	0.437	0.997
45	KHI45	C1	29.3	119.3	0.8	3.3	2.5	2.9	3.1
		C2	59.0	105.2	0.6	2.6	1.8	2.1	2.5
		C3	88.1	194.4	0.7	3.0	1.7	1.3	2.7
		ΔC	58.9	75.1	-0.1	-0.2	-0.8	-1.5	-0.4
		%ΔC	201.2	62.9	-11.4	-6.9	-32.2	-53.5	-14.2
		b	29.4	37.5	-0.1	-0.1	-0.4	-0.8	-0.2
		R^2	0.999	0.613	0.229	0.107	0.872	0.999	0.515
46	KHI46	C1	16.1	47.8	0.8	2.0	1.8	2.4	3.4
		C2	52.6	101.8	0.2	1.5	1.6	2.0	2.7
		C3	99.5	168.1	0.1	0.5	1.0	1.8	3.0
		ΔC	83.4	120.3	-0.7	-1.5	-0.8	-0.6	-0.4
		%ΔC	518.4	251.4	-84.0	-75.0	-43.7	-25.3	-11.7
		b	41.7	60.1	-0.4	-0.8	-0.4	-0.3	-0.2
		R^2	0.995	0.997	0.767	0.964	0.882	0.936	0.307
47	KHI47	C1	54.6	139.5	0.1	3.2	2.7	1.9	2.9
		C2	95.1	135.7	3.7	4.3	1.9	1.6	2.6
		C3	99.6	169.7	1.9	5.0	1.3	1.1	2.5
		ΔC	45.0	30.2	1.8	1.8	-1.4	-0.8	-0.5
		%ΔC	82.3	21.6	1431.7	57.9	-50.4	-42.7	-15.7
		b	22.5	15.1	0.9	0.9	-0.7	-0.4	-0.2
		R^2	0.824	0.657	0.244	0.984	0.995	0.996	0.903
48	KHI48	C1	39.0	107.3	1.0	4.0	2.7	2.7	3.2
		C2	69.1	66.6	0.4	2.5	2.3	2.0	2.8
		C3	84.2	159.2	1.4	6.8	1.3	2.7	2.8
		ΔC	45.2	51.9	0.4	2.8	-1.4	0.0	-0.4
		%ΔC	115.9	48.3	40.1	70.0	-50.3	-0.7	-11.5
		b	22.6	25.9	0.2	1.4	-0.7	0.0	-0.2
		R^2	0.965	0.313	0.147	0.412	0.928	0.001	0.750
49	KHI49	C1	32.0	117.9	1.0	5.3	2.0	2.7	2.7
		C2	39.9	113.6	10.0	9.0	1.3	2.1	2.3
		C3	90.5	213.8	4.5	7.0	1.2	1.9	2.6
		ΔC	58.5	95.9	3.5	1.8	-0.8	-0.8	-0.1
		%ΔC	182.8	81.4	331.6	33.3	-41.4	-30.9	-2.5
		b	33.3	48.0	1.7	0.9	-0.4	-0.4	-0.1
		R^2	0.873	0.717	0.146	0.218	0.794	0.936	0.022
50	KHI50	C1	16.7	59.4	0.5	3.3	3.4	2.1	4.1
		C2	55.6	99.0	0.0	0.0	2.5	2.1	3.3
		C3	70.3	79.0	2.3	2.6	1.7	2.1	1.7
		ΔC	53.6	19.6	1.8	-0.7	-1.7	0.0	-2.4
		%ΔC	320.1	33.0	340.1	-20.0	-49.3	0.0	-57.8
		b	26.8	9.8	0.9	-0.3	-0.8	0.0	-1.2
		R^2	0.937	0.245	0.543	0.036	0.999	0.000	0.972
51	KHI51	C1	29.7	105.0	1.1	4.8	2.6	3.6	3.5

		C2	53.8	103.8	0.3	2.0	1.9	3.1	3.2
		C3	95.7	238.4	0.4	1.9	1.1	1.6	1.9
		ΔC	66.0	133.4	-0.7	-2.9	-1.5	-2.1	-1.6
		%ΔC	222.6	127.0	-63.0	-60.0	-58.3	-56.8	-46.1
		b	33.0	66.7	-0.3	-1.4	-0.7	-1.0	-0.8
		R^2	0.977	0.743	0.641	0.776	0.995	0.930	0.893
52	KHI52	C1	25.8	85.7	1.0	4.3	2.3	2.7	3.5
		C2	40.7	20.5	1.3	2.2	1.9	2.3	3.2
		C3	89.4	173.6	3.6	8.1	1.0	2.2	2.7
		ΔC	63.6	87.8	2.6	3.9	-1.3	-0.6	-0.8
		%ΔC	246.1	102.5	251.7	90.6	-56.5	-20.5	-24.0
		b	31.8	43.9	1.3	1.9	-0.6	-0.3	-0.4
		R^2	0.914	0.327	0.810	0.413	0.939	0.942	0.993
53	KHI53	C1	28.7	95.7	0.6	4.0	2.1	3.6	3.8
		C2	72.0	51.4	1.1	2.6	2.0	2.8	3.0
		C3	84.5	216.8	0.9	3.2	1.9	2.3	2.8
		ΔC	55.8	121.2	0.2	-0.8	-0.3	-1.3	-1.0
		%ΔC	194.3	126.7	32.9	-20.0	-13.1	-37.2	-27.4
		b	27.9	60.6	0.1	-0.4	-0.1	-0.7	-0.5
		R^2	0.908	0.501	0.213	0.344	0.999	0.993	0.899
54	KHI54	C1	48.1	123.5	0.9	3.0	1.6	3.0	2.8
		C2	58.9	95.7	9.0	12.5	1.6	2.4	2.6
		C3	94.9	224.0	7.4	14.5	2.2	2.3	2.9
		ΔC	46.8	100.5	6.5	11.5	0.6	-0.6	0.1
		%ΔC	97.3	81.4	690.9	383.3	37.1	-21.7	5.4
		b	28.4	50.3	3.2	5.8	0.3	-0.3	-0.1
		R^2	0.945	0.554	0.576	0.876	0.675	0.822	0.222
55	KHI55	C1	28.5	78.6	0.5	2.8	4.1	2.8	3.2
		C2	60.8	82.8	1.3	3.2	3.6	2.5	2.8
		C3	97.2	150.9	1.8	3.1	1.6	1.4	2.5
		ΔC	68.7	72.3	1.4	0.4	-2.5	-1.5	-0.7
		%ΔC	241.2	92.0	297.1	14.3	-60.7	-51.6	-22.9
		b	34.4	36.2	0.7	0.2	-1.3	-0.7	-0.4
		R^2	0.999	0.794	0.980	0.669	0.911	0.899	0.985
56	KHI56	C1	22.2	81.4	0.1	1.8	2.6	3.0	4.4
		C2	65.8	80.6	0.3	0.5	2.5	2.8	4.0
		C3	99.1	186.4	2.2	2.7	1.3	1.5	3.2
		ΔC	76.9	105.0	2.1	1.0	-1.3	-1.5	-1.3
		%ΔC	346.6	129.0	3249.1	54.3	-49.3	-50.8	-28.8
		b	38.5	52.5	1.1	0.5	-0.6	-0.8	-0.6
		R^2	0.994	0.745	0.830	0.185	0.824	0.888	0.971
57	KHI57	C1	20.8	35.0	0.6	3.2	1.3	2.7	3.2
		C2	48.6	80.3	1.4	2.7	1.0	2.0	3.0
		C3	60.4	138.6	2.5	5.8	1.3	2.5	2.9

		ΔC	39.6	103.6	1.9	2.6	0.0	-0.2	-0.3
		%ΔC	189.9	296.1	328.7	83.2	-1.2	-7.4	-9.9
		b	19.8	51.8	1.0	1.3	0.0	-0.1	-0.2
		R^2	0.948	0.995	0.994	0.631	0.002	0.076	0.992
58	KHI58	C1	46.2	139.8	0.7	3.5	2.6	3.1	3.5
		C2	56.0	107.0	0.7	1.7	1.8	2.8	2.9
		C3	94.1	185.0	0.1	0.2	1.0	2.3	2.7
		ΔC	47.8	45.2	-0.7	-3.3	-1.6	-0.8	-0.8
		%ΔC	103.4	32.4	-90.0	-94.3	-62.0	-26.5	-22.2
		b	23.9	22.6	-0.3	-1.7	-0.8	-0.4	-0.4
		R^2	0.896	0.334	0.755	0.996	0.999	0.971	0.934
59	KHI59	C1	52.4	157.8	0.8	3.8	3.5	2.6	3.4
		C2	78.3	231.4	4.6	5.1	2.6	1.8	3.1
		C3	99.6	316.5	4.6	2.9	1.5	3.0	2.2
		ΔC	47.2	158.8	3.8	-0.8	-2.0	0.4	-1.2
		%ΔC	90.0	100.6	492.0	-21.9	-57.7	14.2	-34.4
		b	23.6	79.4	1.9	-0.4	-1.0	0.2	-0.6
		R^2	0.997	0.998	0.747	0.137	0.995	0.099	0.932
60	KHI60	C1	26.1	119.0	0.1	2.6	2.1	2.8	3.3
		C2	44.5	68.2	1.0	2.2	1.9	2.3	3.0
		C3	81.0	159.7	0.3	1.3	1.2	2.5	2.8
		ΔC	54.9	40.7	0.2	-1.4	-0.8	-0.3	-0.5
		%ΔC	210.5	34.2	272.9	-52.4	-41.1	-9.3	-16.5
		b	27.5	20.3	0.1	-0.7	-0.4	-0.1	-0.3
		R^2	0.965	0.197	0.043	0.954	0.888	0.286	0.996
61	KHI61	C1	24.8	77.2	0.4	2.3	2.2	3.4	3.5
		C2	37.4	83.0	1.3	1.7	1.6	2.9	3.4
		C3	94.2	263.4	0.7	2.7	1.1	1.4	2.2
		ΔC	69.4	186.2	0.2	0.4	-1.0	-2.0	-1.3
		%ΔC	279.8	241.3	46.7	18.9	-47.8	-57.6	-38.2
		b	34.7	93.1	0.1	0.2	-0.5	-1.0	-1.2
		R^2	0.880	0.774	0.050	0.191	0.994	0.932	0.999
62	KHI62	C1	12.9	34.0	0.4	2.3	2.0	2.0	3.1
		C2	50.1	46.5	0.5	1.1	1.5	1.5	2.5
		C3	77.2	80.6	1.7	5.5	1.5	1.7	2.7
		ΔC	64.3	46.6	1.3	3.2	-0.6	-0.3	-0.4
		%ΔC	498.8	137.0	300.2	143.8	-28.5	-16.3	-12.8
		b	32.1	23.3	0.7	1.6	-0.3	-0.2	-0.2
		R^2	0.992	0.933	0.790	0.501	0.739	0.355	0.417
63	KHI63	C1	22.0	92.0	0.6	2.0	3.2	2.3	3.5
		C2	53.7	165.5	0.4	1.7	2.3	1.8	2.8
		C3	99.8	312.0	0.7	2.3	1.1	1.1	3.0
		ΔC	77.8	220.0	0.1	0.3	-2.1	-1.2	-0.5
		%ΔC	353.8	239.1	13.8	15.0	-66.4	-53.5	-13.6

		b	38.9	110.0	0.1	0.2	-1.1	-0.6	-0.2
		R^2	0.989	0.965	0.095	0.228	0.994	0.998	0.472
64	KHI64	C1	7.5	30.0	0.7	4.3	2.4	2.5	4.0
		C2	55.9	73.4	5.8	5.5	2.4	2.0	3.5
		C3	90.3	203.0	8.5	12.5	2.2	1.6	2.6
		ΔC	82.8	173.0	7.8	8.3	-0.2	-1.0	-1.4
		%ΔC	1104.0	576.7	1181.6	194.1	-9.1	-37.9	-34.4
		b	48.9	100.0	3.9	4.1	-0.1	-0.5	-0.7
		R^2	0.981	0.961	0.968	0.861	0.914	0.999	0.971
65	KHI65	C1	28.8	88.2	1.6	7.5	2.8	3.3	3.3
		C2	55.5	129.1	3.3	6.0	2.2	2.6	3.1
		C3	76.9	157.6	1.6	3.9	1.2	1.0	2.6
		ΔC	48.1	69.4	-0.1	-3.6	-1.6	-2.3	-0.7
		%ΔC	167.3	78.6	-4.5	-48.0	-56.8	-70.0	-22.4
		b	24.1	34.7	-0.1	-1.8	-0.8	-1.2	-0.4
		R^2	0.996	0.990	0.002	0.991	0.983	0.961	0.937
66	KHI66	C1	8.7	27.0	0.5	1.1	3.4	2.8	3.2
		C2	45.2	87.3	0.4	1.3	3.1	2.2	2.8
		C3	91.1	123.7	3.1	4.9	2.3	2.2	2.6
		ΔC	82.4	96.7	2.6	3.8	-1.1	-0.6	-0.6
		%ΔC	945.2	358.1	572.3	337.0	-32.3	-20.6	-19.3
		b	41.2	48.4	1.3	1.9	-0.6	-0.3	-0.3
		R^2	0.996	0.980	0.737	0.775	0.942	0.706	0.979
67	KHI67	C1	13.6	59.0	0.5	3.0	1.9	2.8	3.7
		C2	31.7	68.8	1.7	4.5	1.6	2.0	3.3
		C3	96.6	229.7	0.3	1.0	1.4	1.2	2.2
		ΔC	83.0	170.7	-0.2	-2.0	-0.5	-1.7	-1.5
		%ΔC	611.9	289.3	-44.6	-65.8	-25.5	-58.3	-39.9
		b	41.5	85.4	-0.1	-1.0	-0.2	-0.8	-0.7
		R^2	0.905	0.793	0.023	0.321	0.960	0.999	0.928
68	KHI68	C1	4.5	14.0	0.5	3.8	2.1	2.6	2.9
		C2	12.5	26.0	1.4	6.0	1.3	2.4	2.5
		C3	83.5	164.0	0.2	0.6	1.3	1.2	2.6
		ΔC	79.0	150.0	-0.3	-3.2	-0.8	-1.4	-0.3
		%ΔC	1765.7	1071.6	-69.7	-85.3	-37.5	-52.8	-9.4
		b	39.5	75.0	-0.2	-1.6	-0.4	-0.7	-0.1
		R^2	0.826	0.810	0.071	0.341	0.758	0.878	0.422
69	KHI69	C1	17.7	47.5	0.3	0.5	1.9	2.3	3.1
		C2	9.7	4.7	0.4	1.0	1.6	1.9	2.7
		C3	60.5	117.3	0.3	1.2	1.0	3.1	3.2
		ΔC	42.8	69.8	0.0	0.7	-0.9	0.7	0.1
		%ΔC	242.3	146.8	9.9	140.0	-48.1	32.1	4.2
		b	21.4	34.9	0.0	0.4	-0.5	0.4	0.1
		R^2	0.614	0.377	0.094	0.942	0.972	0.372	0.074

70	KHI70	C1	6.5	20.5	1.0	4.8	1.6	2.4	3.3
		C2	7.8	18.0	1.6	6.4	1.5	2.2	2.9
		C3	100.0	185.7	0.5	1.5	1.7	2.2	3.0
		ΔC	93.5	165.2	-0.5	-3.3	0.1	-0.2	-0.3
		%ΔC	1436.6	805.7	-50.3	-68.4	6.2	-8.2	-9.9
		b	46.7	82.6	-0.3	-1.6	0.1	-0.1	-0.2
		R^2	0.761	0.739	0.226	0.429	0.242	0.544	0.537
71	KHI71	C1	47.5	124.0	1.4	5.3	1.8	2.5	3.6
		C2	41.3	52.9	2.7	6.3	1.3	2.2	3.1
		C3	99.9	222.8	0.6	1.8	1.7	1.0	2.4
		ΔC	52.4	98.8	-0.9	-3.5	-0.1	-1.5	-1.2
		%ΔC	110.5	79.7	-60.7	-65.7	-7.5	-60.1	-33.5
		b	26.2	49.4	-0.4	-1.7	-0.1	-0.8	-0.6
		R^2	0.663	0.335	0.163	0.531	0.071	0.902	0.990
72	KHI72	C1	41.6	112.1	1.2	5.8	2.2	2.3	3.2
		C2	49.7	130.5	1.0	3.5	1.8	2.0	2.7
		C3	98.0	243.5	0.4	2.0	1.0	2.5	3.0
		ΔC	56.4	131.4	-0.7	-3.8	-1.2	0.2	-0.2
		%ΔC	135.7	117.3	-63.0	-66.1	-55.5	11.0	-5.0
		b	28.2	65.7	-0.4	-1.9	-0.6	0.1	-0.1
		R^2	0.856	0.853	0.924	0.989	0.967	0.299	0.138
73	KHI73	C1	45.0	131.9	0.3	2.6	1.9	2.0	2.7
		C2	26.1	41.3	0.7	2.0	1.1	1.5	2.4
		C3	85.0	181.7	0.8	2.8	2.0	2.8	3.0
		ΔC	39.9	49.8	0.5	0.2	0.1	0.8	0.3
		%ΔC	88.7	37.7	195.4	6.7	3.5	38.9	9.6
		b	20.0	24.9	0.3	0.1	0.1	0.4	0.1
		R^2	0.442	0.122	0.920	0.043	0.004	0.372	0.165
74	KHI74	C1	13.7	32.6	0.9	4.3	1.5	2.7	4.1
		C2	12.4	3.0	0.3	1.0	1.0	2.3	3.4
		C3	67.0	68.5	0.4	1.5	2.1	3.6	3.6
		ΔC	53.3	35.9	-0.5	-2.8	0.6	0.9	-0.5
		%ΔC	388.1	110.1	-57.3	-65.0	38.3	33.9	-12.4
		b	26.6	18.0	-0.3	-1.4	0.3	0.5	-0.3
		R^2	0.731	0.296	0.600	0.622	0.273	0.429	0.435
75	KHI75	C1	47.4	149.4	0.3	1.7	2.6	3.1	3.4
		C2	27.3	48.5	0.8	2.5	1.8	2.6	2.7
		C3	96.0	244.9	1.0	4.4	1.8	4.3	3.0
		ΔC	48.6	95.5	0.8	2.7	-0.8	1.2	-0.4
		%ΔC	102.6	63.9	305.9	164.0	-29.2	37.9	-11.1
		b	24.3	47.8	0.4	1.4	-0.4	0.6	-0.2
		R^2	0.473	0.236	0.963	0.952	0.753	0.472	0.342
76	KHI76	C1	22.1	51.0	0.9	5.8	4.0	4.0	4.0
		C2	6.1	4.5	0.7	2.5	3.9	3.3	4.0

		C3	100.0	161.0	0.1	0.3	2.5	4.5	3.0
		ΔC	77.9	110.0	-0.8	-5.5	-1.5	0.5	-1.0
		%ΔC	352.8	215.7	-87.6	-95.7	-37.5	12.5	-25.0
		b	39.0	55.0	-0.4	-2.8	-0.7	0.3	-0.5
		R^2	0.601	0.468	0.918	0.989	0.824	0.158	0.750
77	KHI77	C1	34.4	72.1	1.6	7.8	1.9	2.6	3.7
		C2	14.4	16.2	1.5	5.5	1.6	2.0	2.8
		C3	88.8	169.4	1.2	4.9	1.0	2.2	3.2
		ΔC	54.3	97.3	-0.4	-2.9	-0.9	-0.5	-0.5
		%ΔC	157.8	135.0	-25.3	-36.8	-47.7	-17.9	-13.8
		b	27.2	48.6	-0.2	-1.4	-0.5	-0.2	-0.3
		R^2	0.499	0.394	0.875	0.899	0.986	0.550	0.362
78	KHI78	C1	9.6	13.5	0.8	3.0	2.2	2.7	4.0
		C2	2.9	3.0	0.4	0.3	1.5	2.2	3.3
		C3	58.5	161.8	1.5	4.0	2.4	4.3	2.8
		ΔC	48.9	148.3	0.8	1.0	0.2	1.7	-1.3
		%ΔC	506.7	1098.1	103.3	33.3	8.3	62.6	-31.6
		b	24.4	74.1	0.4	0.5	0.1	0.8	-0.6
		R^2	0.649	0.697	0.477	0.066	0.041	0.556	0.993
79	KHI79	C1	23.4	57.8	0.3	1.0	1.9	2.1	3.5
		C2	7.2	2.2	0.4	1.7	1.4	1.9	3.0
		C3	44.2	83.8	0.7	2.6	1.9	3.0	2.8
		ΔC	20.9	25.9	0.5	1.6	0.0	0.9	-0.7
		%ΔC	89.3	44.8	186.8	163.3	1.5	44.3	-21.3
		b	10.4	13.0	0.2	0.8	0.0	0.5	-0.4
		R^2	0.316	0.097	0.945	0.989	0.002	0.599	0.966
80	KHI80	C1	43.1	142.5	0.7	2.8	2.0	2.6	3.6
		C2	41.0	61.7	0.7	2.0	1.0	2.0	3.1
		C3	98.7	178.6	0.2	0.9	1.5	3.3	2.9
		ΔC	55.7	36.1	-0.4	-1.9	-0.5	0.7	-0.7
		%ΔC	129.2	25.3	-67.0	-67.3	-23.7	27.3	-19.5
		b	27.8	18.1	-0.2	-0.9	-0.2	0.4	-0.3
		R^2	0.722	0.091	0.608	0.988	0.233	0.309	0.941

C1, C2, and C3 represent breeding cycle 1, 2, and 3, respectively. ΔC is increase due to selection. $\%\Delta C$ is percentage increase due to selection. *b* realized genetic gain per cycle. R^2 coefficient of determination.
